# Cholesterol‐lowering drug targets reduce risk of dementia: Mendelian randomization and meta‐analyses of 1 million individuals

**DOI:** 10.1002/alz.70638

**Published:** 2025-10-08

**Authors:** Liv Tybjærg Nordestgaard, Aimee Hanson, Eleanor Sanderson, Emma Anderson, Venexia Walker, Anne Tybjærg‐Hansen, George Davey Smith, Børge G. Nordestgaard

**Affiliations:** ^1^ Department of Clinical Biochemistry Copenhagen University Hospital – Herlev and Gentofte Herlev Denmark; ^2^ Medical Research Council Integrative Epidemiology Unit Population Health Sciences, University of Bristol Bristol UK; ^3^ Division of Psychiatry University College London London UK; ^4^ Department of Clinical Biochemistry Copenhagen University Hospital – Rigshospitalet Copenhagen Denmark; ^5^ The Copenhagen General Population Study Copenhagen University Hospital – Herlev and Gentofte Herlev Denmark; ^6^ Department of Clinical Medicine Faculty of Health and Medical Sciences University of Copenhagen Copenhagen Denmark; ^7^ The Copenhagen City Heart Study Copenhagen University Hospital – Bispebjerg and Frederiksberg Frederiksberg Denmark

**Keywords:** atherosclerosis, β‐hydroxy β‐methylglutaryl‐CoA reductase, cholesterol, cholesteryl ester transfer protein, dementia, genetics, lipid lowering, lipoproteins, Mendelian randomization, Niemann‐Pick C1‐like 1

## Abstract

**INTRODUCTION:**

We tested whether genetically proxied non‐high‐density lipoprotein cholesterol (non‐HDL‐C)–lowering drug targets reduce risk of all‐cause dementia.

**METHODS:**

We included 1,091,775 individuals from three prospective general population cohorts with individual‐level data and two consortia with summary‐level data. We selected genetic variants within *HMGCR, NPC1L1, PCSK9, ANGPTL4, LPL*, and *CETP* associated with non‐HDL‐C. These variants were used as exposures in Cox regression and one‐ and two‐sample Mendelian randomization. Results were meta‐analyzed.

**RESULTS:**

Meta‐analysis of one‐sample Mendelian randomization odds ratios per 1 mmol/L (39 mg/dL) lower non‐HDL‐C was 0.24 (0.18–0.31) for *HMGCR*, 0.18 (0.12–0.25) for *NPC1L1*, 0.97 (0.70–1.35) for *PCSK9*, 1.66 (0.52–5.36) for *ANGPTL4*, 1.41 (0.63–3.16) for *LPL*, and 0.30 (0.26–0.34) for *CETP*. Cox regression and two‐sample Mendelian randomization results were mostly directionally consistent.

**DISCUSSION:**

Genetic lowering of non‐HDL cholesterol via *HMGCR, NPC1L1*, and *CETP* reduces the risk of dementia. This reflects the effect of lifelong differences in non‐HDL cholesterol on risk of dementia.

**Highlights:**

- Variants in *HMGCR, NPC1L1*, and *CETP* reduce the risk of dementia via non‐high‐density lipoprotein cholesterol (non‐HDL‐C).
- An effect of *PCSK9, ANGPTL4*, and *LPL* variants on dementia risk cannot be excluded.
- This reflects the effect of lifelong lower non‐HDL‐C on risk of dementia.

## 1 BACKGROUND

Dementia is a devastating neurodegenerative disease currently affecting 50 million people worldwide.^1^ Although efforts to treat and prevent dementia have been ongoing for years, little progress has been made.^1^ However, recent research is pointing toward a shared pathogenesis between dementia and atherosclerotic cardiovascular disease.^2^ Importantly, several shared modifiable risk factors have been suggested, including blood lipids like low‐density‐lipoprotein (LDL) cholesterol,^3^ triglycerides,^4^ and non‐high‐density lipoprotein (non‐HDL) cholesterol.^5^ The biological mechanism linking these risk factors to dementia could be through atherosclerosis and consequently through the development of strokes and microinfarcts in the brain.^2^ Therefore, investigating already available drugs aimed at treating and preventing atherosclerotic cardiovascular disease may aid in the search for new prevention strategies for dementia.

Lipid‐lowering drugs have been effective at reducing the risk of atherosclerotic cardiovascular disease.^6^ The most well‐known lipid‐lowering drug target so far is the β‐hydroxy β‐methylglutaryl‐CoA (HMG‐CoA) reductase (HMGCR) that catalyzes a vital step in the biosynthesis of cholesterol and is the target for statins.^7^ Other, more recent drugs include ezetimibe, which blocks the critical mediator of intestinal cholesterol absorption, the Niemann‐Pick C1‐like 1 (NPC1L1) protein,^8^ and alirocumab/evolocumab, which inhibit proprotein convertase subtilisin/kexin type‐9 (PCSK9), which binds to the LDL receptor (LDLR) and targets it for lysosomal degradation in cells.^9^ Lipoprotein lipase (LPL) is a protein that hydrolyzes triglycerides in triglyceride‐rich lipoproteins, and its deficiency results in severe hypertriglyceridemia.^10^ Even though gene therapy for LPL deficiency lowers triglyceride concentrations in patients with familial chylomicronemia syndrome, it is no longer marketed.^10^ Drugs against inhibitors of LPL are currently under development. These include drugs targeting angiopoietin‐like 4 (ANGTPL4), angiopoietin‐like 3 (ANGTPL3), and apolipoprotein C‐III (apo C‐III).^10^ Other lipid‐lowering drugs currently in development include inhibitors of cholesteryl ester transfer protein (CETP),^11^ even though failure of previous trials would indicate that the successful development of CETP inhibitors is less likely.^12^ CETP is a protein that facilitates the transfer of cholesterol from HDL to triglyceride‐rich lipoproteins and LDL in exchange for triglycerides,^11^ and inhibition of CETP results in a favorable lipid profile with lower non‐HDL cholesterol.

Mendelian randomization is a method that takes advantage of the random assortment of alleles during reproduction^13^, ^14^ and is therefore largely unconfounded by behavioral and environmental confounders of observational studies. Using genetic evidence, such as Mendelian randomization, in the selection of drug targets has been estimated to double the success rate in clinical trial development.^15^ Therefore, it is an appropriate method for identifying potential targets for drug repurposing. Key assumptions for Mendelian randomization studies are (1) the genetic variants strongly associate with the exposure of interest, (2) there are no unmeasured confounders of the associations between genetic variants and the outcome, and (3) the genetic variants affect the outcome only through their effect on the exposure of interest.^13^, ^14^

We examined the effect of genetic proxies for six non‐HDL cholesterol–lowering drug targets on the risk of vascular dementia, unspecified dementia, Alzheimer's disease (AD), and all‐cause dementia. Ischemic heart disease was used as a positive control. We applied two different approaches using three prospective cohorts (Copenhagen General Population Study [CGPS], Copenhagen City Heart Study [CCHS], UK Biobank) with individual‐level data: (1) Cox regression analysis with unweighted allele scores and (2) one‐sample Mendelian randomization. We included two further large cohorts (FinnGen and Global Lipids Genetics Consortium [GLGC]) with summary‐level data and used all five cohorts for two‐sample Mendelian randomization. Finally, we performed meta‐analyses on the results from Cox regression and Mendelian randomization analyses.

## 2 METHODS

### 2.1 Study populations

#### 2.1.1 CGPS and the CCHS

CGPS was initiated in 2003 with ongoing enrollment. Individuals were selected based on the national Danish Civil Registration System to reflect the adult Danish population aged 20 to 100 years.^16^ Data were obtained from a questionnaire, a physical examination, and a blood sample. The CCHS was initiated in 1976 through 1978, with follow‐up examinations in 1981 to 1983, 1991 to 1994, and 2001 to 2003;^16^ we used data from the 1991 to 1994 examination, for which DNA was available. End of follow‐up for this study was December 31, 2021. Participants were recruited and examined as in the CGPS. We included 122,940 individuals from the CCHS and CGPS. The CCHS and CGPS had participation rates of 61% and 43%, respectively.^17^, ^18^ Participants were White and of Danish descent. The present study protocol was approved before the study started by the steering committees of the CGPS and the CCHS, by institutional review boards, and by the Danish data protection agency. The overall studies were approved by local Danish ethical committees, adhered to the principles of the Declaration of Helsinki, and all participants provided written informed consent.

**RESEARCH IN CONTEXT**

1. **Systematic review**: The authors reviewed the literature using traditional (e.g., PubMed) sources and meeting abstracts and presentations. While the effect of lipid‐lowering drug targets on risk of dementia has been studied to some extent, the effect of genetic variants in these drug target genes on risk of dementia is less well studied. However, the relevant studies are appropriately cited.
2. **Interpretation**: Our findings lead to an understanding of the potential role of lipid‐lowering drug targets in dementia prevention.
3. **Future directions**: This article proposes a framework for further investigation of lipid‐lowering drug targets as a prevention strategy in dementia. Examples include clinical trials with > 20 years of follow‐up and repetition of the current study when more follow‐up time is available in the cohorts used.

#### 2.1.2 UK Biobank

The UK Biobank is a population‐based health research resource consisting of ≈ 500,000 people, aged between 38 and 73 years, who were recruited between the years 2006 and 2010 from across the UK.^19^ End of follow‐up for this study was March 31, 2022. Particularly focused on identifying determinants of human diseases in middle‐aged and older individuals, participants provided a range of information via questionnaires and interviews; anthropometric measures, blood pressure readings, and samples of blood were also taken. A full description of the study design, participants, and quality control (QC) methods has been described in detail previously.^19^, ^20^ We included 383,572 individuals of European ancestry in the analysis. Access to information from participants was approved by the Patient Information Advisory Group (PIAG) from England and Wales. All participants provided electronic written informed consent for the study. Our study was conducted under UK Biobank application number 81499.

#### 2.1.3 FinnGen

FinnGen is a large research collaboration launched in Finland in 2017 with the scope to collect and analyze genomic and health data from 500,000 Finnish biobank participants.^21^ FinnGen includes 9 Finnish biobanks, research institutes, universities, and university hospitals; 13 international pharmaceutical industry partners; and the Finnish Biobank Cooperative (FINBB).^22^ The project uses data from the nationwide longitudinal health register collected since 1969 from every resident in Finland. It produces near‐complete genome variant data from all 500,000 participants using genome‐wide association study (GWAS) genotyping and imputation. The FinnGen data release (F10) from December 18, 2023, was included in this study (*n* = 412, 181). Only summary‐level data were available. There was no sample overlap with any of the other cohorts.

#### 2.1.4 GLGC

GLGC is a worldwide collaboration dedicated to investigating the genetics of lipid traits.^23^ The GLGC GWAS is a meta‐analysis of 37 studies containing 173,082 individuals of European descent. The dataset not including UK Biobank was used in this study. For more information, see Willer et al.^23^ Only summary‐level data were available. There was no sample overlap with any of the other cohorts.

### 2.2 Covariates and laboratory analyses in individual‐level data

Sex and age were determined by the central personal register number in the CCHS andCGPS and by date of birth and genetic sex in the UK Biobank.

In the CCHS and CGPS, non‐fasting plasma total cholesterol was measured using standard hospital assays at the time of study entry. LDL cholesterol was calculated using the Friedewald equation when plasma triglycerides were ≤ 4.0 mmol/L (155 mg/dL) and otherwise measured directly. Non‐fasting plasma triglycerides were measured using standard hospital assays with enzymatic methods at the time of study entry. In the UK Biobank, total plasma cholesterol, LDL cholesterol, and triglycerides were measured by standard enzymatic methods. In CCHS and CGPS and UK Biobank, non‐HDL cholesterol was total cholesterol minus HDL cholesterol measured by a direct assay. In CCHS, CGPS, and UK Biobank, blood samples were taken at random irrespective of time since and content of the last meal.^19^, ^24^ Missing values for laboratory analyses were 1% to 4% in CCHS and CGPS and 5% to 13% in the UK Biobank. Missing values were not imputed.

An ABI PRISM 7900HT Sequence Detection System (Applied Biosystems Inc.) and TaqMan‐based assays were used to genotype the selected variants in the CCHS and CGPS, and Hardy–Weinberg equilibrium was calculated. When results from TaqMan were unclear Sanger sequencing was used to validate results. For the UK Biobank, we extracted relevant information for the same genetic variants from the genotype data release (July 2023), genome build GRCh37.

### 2.3 Instrument selection

Genetic instruments were chosen based on data availability in the CCHS and CGPS. The variants that have been genotyped in the CCHS and CGPS are well‐established variants from targeted or GWASs. These genetic variants represent independent loci, show strong associations between the genotype and the lipid trait, and map to the drug target genes. They represent the most important drug targets currently in use (HMGCR, NPC1L1, and PCSK9), targets in current drug trials (ANGPTL4 and CETP), or targets that have previously been marketed (LPL).

In total, one, one, three, one, one, and three genetic variants were used for *HMGCR, NPC1L1, PCSK9, ANGPTL4, LPL*, and *CETP*, respectively. They were either combined into scores when consisting of more than one variant (by counting the number of relevant lipid‐lowering alleles of the variant[s] for each individual) or used as individual variants in the Cox regression analyses. One score per gene was generated. In one‐sample Mendelian randomization analyses, variants were used as individual instruments. Importantly, none of the variants were originally discovered in either the CCHS, CGPS, or UK Biobank. An *R* ^2^ threshold of 0.05 for linkage disequilibrium was used to exclude correlated genetic variants.

**FIGURE 1:**
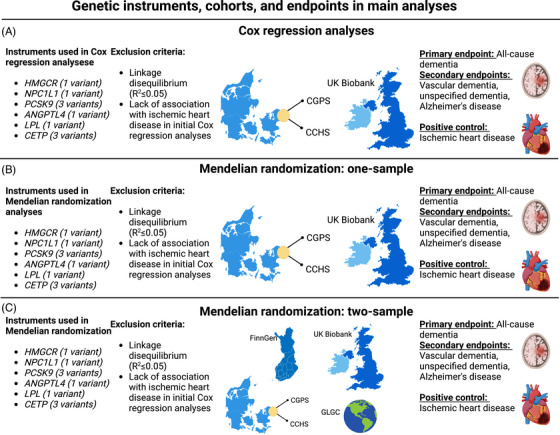
Genetic instruments, cohorts, and endpoints in main analyses. A, Cox regression analyses in the CCHS + CGPS and UK Biobank. Selected variants were available in both the CCHS + CGPS and UK Biobank. Variants were excluded from main analyses if they were in linkage disequilibrium (threshold*r* ^2^ ≤ 0.05) or if there was a lack of association between the allele score and the positive control IHD in Cox regression analyses. B, The same variants were included in one‐sample Mendelian randomization analyses in the CCHS + CGPS and UK Biobank. C, The same variants were included in two‐sample Mendelian randomization analyses in the CCHS + CGPS, UK Biobank, FinnGen, and GLGC. *ANGPTL4*, angiopoietin like 4; *CETP*, cholesteryl ester transfer protein; CCHS, Copenhagen City Heart Study; CGPS, Copenhagen General Population Study; GLGC, Global Lipids Genetics Consortium; *HMGCR*, β‐hydroxy β‐methylglutaryl‐CoA reductase; IHD, ischemic heart disease; *LPL*, lipoprotein lipase; *NPC1L1*, Nieman pick C1‐like 1; PCSK9, proprotein convertase subtilisin/kexin type 9; SD, standard deviation. This figure was created with Biorender.com.

The *HMGCR* variant was chosen because it is a common variant (minor allele frequency > 5%) and is strongly associated with LDL cholesterol (*P* = 6 × 10^−116^ in the GLGC), a large GWAS of lipid traits.^25^ The *NPC1L1* variant was chosen because it is a common variant (minor allele frequency > 5%) with a strong association to LDL cholesterol (*P* = 2 × 10^−20^ in the GLGC).^8^, ^25^ The *PCSK9* variants were selected because they are robustly associated with PCSK9 and LDL cholesterol concentrations in different populations of European ancestry^26^ (*P* = 1 × 10^−124^, 3 × 10^−101^, and 5 × 10^−324^ in GLGC). The instruments for triglyceride‐lowering drugs were all located in genes with known important function in triglyceride hydrolysis. The *LPL* variant is a well‐known amino‐acid changing variant with an effect on LPL activity and triglyceride levels (*P* = 8 × 10^−43^).^23^, ^25^ The *ANGPTL4* variant was chosen because it was one of the first variants in ANGPTL4 to be re‐sequenced in a large population and because it is strongly associated with plasma triglycerides (*P* = 5 × 10^−324^).^25^, ^27^ For *CETP* the core promotor, coding exons, and exon–intron boundaries were sequenced in individuals with the lowest and highest 2% HDL cholesterol in the CCHS to identify variants associated with extreme HDL cholesterol levels in the general population. Variants with a minor allele frequency > 1% were then genotyped in 102,607 individuals in CCHS and CGPS^11^ (*P* for selected variants in *CETP* = 6 × 10^−8^, *P* = 4 × 10^−156^ and 2 ×^10‐31^ in GLGC). For a detailed description of all available variants in CCHS and CGPS, including selected variants, see Table S1 in supporting information, and for a flowchart of instrument selection, see Figure 1. See Table S2 in supporting information for *P* values for association with non‐HDL cholesterol in GLGC for each variant.

### 2.4 Choice of lipid trait used as exposure in main and sensitivity analyses

As the variants in *HMGCR*, *NPC1L1*, and *PCSK9* mainly affect levels of LDL cholesterol, and variants in *ANGPTL4* and *LPL* mainly affect levels of triglycerides (as a marker of triglyceride‐rich lipoproteins such as very‐low‐density lipoprotein and intermediate‐density lipoproteins)^28^, ^29^ initial analyses have been performed using LDL cholesterol and triglycerides as exposures (instrumented by variants in these drug target genes). Genetic variation in *CETP* affects levels of HDL cholesterol, but because HDL cholesterol has never been shown to causally affect the risk of atherosclerosis^11^, ^30^ HDL cholesterol has not been used as an exposure.^31^ Instead, LDL cholesterol was used as an exposure. For comparability purposes non‐HDL cholesterol was used in main analyses because this trait includes both atherogenic cholesterol in LDL particles as well as in triglyceride‐rich lipoproteins.

### 2.5 Outcomes

Diagnoses of diseases were according to the International Classification of Disease (ICD). Versions 8 and 10 were used in CCHS and CGPS (version 9 was never implemented in Denmark), while versions 9 and 10 were used in the UK Biobank, and versions 8, 9, and 10 were used in FinnGen. Vascular dementia was defined as ICD‐9 290.4 and ICD‐10 F01; unspecified dementia was defined as ICD‐8 290.09, 290.18, and 290.19, ICD‐9 294.2, and ICD‐10 F03; and AD was defined as ICD‐8 290, ICD‐9 331.0, and ICD‐10 F00 and G30 (Table S3 in supporting information). All‐cause dementia included vascular dementia, unspecified dementia, and AD in CCHS and CGPS and in UK Biobank. In FinnGen all‐cause dementia included vascular dementia, unspecified dementia, AD, dementia in other diseases classified elsewhere (ICD‐10 F02), or use of anticholinesterases or other dementia drugs (KELA reimbursement code 307 and medicine purchase ATC‐N06D).^21^ In Denmark, cerebrospinal fluid biomarkers and imaging studies are performed on most of the patients in the clinical setting, and AD versus vascular dementia diagnoses are based on a combination of these findings with clinical symptoms.^32^ As a positive control, we included ischemic heart disease (IHD), which was defined as ICD‐8 410 to 414, ICD‐9 410 to 414, and ICD‐10 I20 to I25, including myocardial infarction (MI; ICD‐8 410, ICD‐9 410, and ICD‐10 I21 to I22). To verify the validity of the diagnoses in CCHS and CGPS and UK Biobank apolipoprotein E (*APOE*) genotype frequencies were calculated (Table S4 in supporting information). In sensitivity analyses we categorized all‐cause dementia cases in CCHS and CGPS and UK Biobank according to whether they had zero, one, or two copies of the *APOE* ɛ4 allele.

### 2.6 Statistical analyses

Data were analyzed using Stata SE 17.0 and R version 4.1.0. In our main analyses, all estimates are reported as per 1 mmol/L (39 mg/dL) lower non‐HDL cholesterol. In our sensitivity analyses, estimates are reported as per 1 mmol/L lower LDL cholesterol or per halving in triglycerides. Because plasma triglyceride concentrations are not normally distributed, their values were log‐transformed (log2_trig = ln[TRIG]/ln[2]) before inclusion in further analyses; accordingly, we used a halving of triglyceride concentrations rather than a 1 mmol/L (88 mg/dL) lower concentration.

In CCHS and CGPS and UK Biobank, we generated an unweighted allele score for each drug target based on the selected variants, that is, by counting the number of relevant lipid‐lowering alleles of the variant(s) for each individual. To transform the effect of unweighted allele scores to a clinically interpretable scale, each allele score was multiplied by 1/allele score specific effect on relevant lipid trait in CCHS and CGPS and UK Biobank, respectively. These were used in Cox regression analyses.

To examine the possible causal effect of lifelong lower non‐HDL cholesterol caused by lipid‐lowering drug targets on risk of dementia and IHD, we used Cox proportional hazard models with age as time scale and delayed entry at examination (left truncation). Individuals diagnosed with either dementia or IHD before study entry were excluded, and those dying or emigrating during follow‐up were censored at their death or emigration date. For Cox regression models, proportionality of hazards over time was assessed by plotting the negative natural logarithm of survival versus the natural logarithm of analysis time. We adjusted for age and sex in CCHS and CGPS and for age, sex, and first 10 principal components in UK Biobank. In initial exploratory analyses, allele scores for other potential drug targets (*APOC3* and *ANGPTL3*) that did not associate robustly with risk of IHD were excluded from further analyses. The rationale behind this is that if there is insufficient power to show an effect on IHD (for which we have substantially more power [25,000 events]), results from analyses investigating a much less well‐powered endpoint (9000 events) will likely result in unclear inference. Results from Cox regression were ultimately combined in a meta‐analysis for each outcome using fixed‐effect models.^33^

To examine the possible causal effect between genetic deficiency of lipid‐lowering drug targets using Mendelian randomization, we used two‐stage predictor substitution estimators with second stage logistic regression from the OneSampleMR package (https://cran.r‐project.org/web/packages/OneSampleMR/OneSampleMR.pdf) to fit a first‐stage model of the exposure on the instruments, obtained predicted values of exposures (lipid levels), and then fit a second‐stage model of the outcome regressed on the predicted values of the exposure. This estimates an odds ratio for a change in risk of outcome per unit change in genetically lower non‐HDL cholesterol. We adjusted for age and sex in CCHS and CGPS and age, sex, and first 10 principal components in UK Biobank. All genetic variants were used as individual instruments in these analyses. We performed the analyses both including all available variants in CCHS and CGPS and using only variants with *R* ^2^ ≤ 0.05. Results from Mendelian randomization analyses were then combined in a meta‐analysis for each outcome using fixed‐effect models.^33^

Summary data for two‐sample Mendelian randomization were derived using logistic regression and adjusted for age and sex in CCHS and CGPS and for age, sex, and first 10 principal components in UK Biobank to generate outcome data. We accessed summary data from GLGC (exposure data; https://www.lipidgenetics.org/#data‐downloads‐title) and FinnGen (outcome data; https://www.finngen.fi/en/access_results). GLGC summary data were adjusted for age, age^2^, principal components of ancestry, and any necessary study‐specific covariates.^34^ FinnGen summary data were adjusted for sex, age, genotyping batch, and ten PCs.^22^ The TwoSampleMR R package (https://mrcieu.github.io/TwoSampleMR/) was used for analyses.^35^ In the main analyses, results from Wald ratio analyses were used for exposures with one variant, and results from weighted median analyses were used for exposures with at least three variants. In sensitivity analyses, weighted mode, inverse variance weighted, simple mode, and Mendelian randomization Egger were used for exposures with at least three variants.

To assess the first assumption of Mendelian randomization (see section 1 for the assumptions of Mendelian randomization) in one‐sample Mendelian randomization, we calculated *F* statistics for each of the unweighted allele scores and individual genetic variants included in the study. The third assumption was falsified by investigating the association of the genetic variants included in the study with levels of other lipids and apolipoprotein. Further, for drug targets with more than one genetic instrument, Sargan statistics were calculated using the ivreg package (https://cran.r‐project.org/web/packages/ivreg/vignettes/ivreg.html). To test the first assumption, in two‐sample Mendelian randomization, *F* statistics for individual instruments were calculated, and to falsify the third assumption, tests of pleiotropy and heterogeneity were performed.

## 3 RESULTS

Baseline characteristics of study participants in the CCHS and CGPS and the UK Biobank are shown in Table 1. Among 122,940 individuals from the CCHS and CGPS, 5743 developed all‐cause dementia (including 620 vascular dementia, 3107 unspecified dementia, and 3277 AD cases), and 17,129 developed IHD between the start of the Danish Patient Registry (January 1, 1977) and the end of follow‐up (December 31, 2021). Among 383,572 individuals from the UK Biobank, 4451 developed all‐cause dementia (including 1222 vascular dementia, 2761 unspecified dementia, and 2320 AD cases), and 21,966 developed IHD between June 1997 and the end of follow‐up (March 31, 2022). Median follow‐up in the CCHS and CGPS was 12 years (range: < 1–46 years). Median follow‐up in the UK Biobank was 13 years (range: < 1–15 years). Data from FinnGen included 412,181 individuals with 19,157 all‐cause dementia cases (including 2667 vascular dementia, 4408 unspecified dementia, and 6145 AD cases) and 69,101 IHD cases. Information on endpoints in FinnGen was collected from the nationwide Finnish Health Registry (since 1969) until the end of follow‐up on December 18, 2023.

**TABLE 1:** Baseline characteristics of individuals by cohort.

	CCHS + CGPS	UK Biobank
No. of individuals, %	122,940	383,572
Women, %	55	54
Age, years	72 (62–81)	72 (64–77)
Current smoking, %	22	10
High alcohol consumption, %	46	21
Triglycerides, mmol/L	1.4 (1.0–2.1)	1.4 (1.0–2.1)
Triglycerides, mg/dL	124 (89–186)	124 (89–363)
Lipid‐lowering therapy, %	11	10
LDL cholesterol, mmol/L	3.2 (2.6–3.8)	3.5 (3.0–4.1)
LDL cholesterol, mg/dL	124 (101–147)	124 (116–159)
Systolic blood pressure, mmHg	140 (127–155)	137 (125–150)
Diastolic blood pressure, mmHg	84 (76–91)	82 (75–89)
Body mass index, kg/m^2^	26 (23–28)	27 (24–30)
Non‐HDL cholesterol, mmol/L	4.0 (3.2–4.8)	4.2 (3.5–5.0)
Non‐HDL cholesterol, mg/dL	155 (124–186)	162 (135–193)
Physical inactivity, %	50	48
Low education, %	16	18

*Note*: Continuous values are given as median (interquartile range). Sex and age were determined by central personal register number in the CCHS + CGPS and by date of birth and genetic sex in the UK Biobank. Smoking was current smoking and self‐reported in all cohorts. Body mass index was calculated as measured weight (kg) divided by measured height in meters squared (m^2^) in all cohorts. High alcohol consumption was > 7/14 units per week for women/men (1 unit = 12 g alcohol, equivalent to one glass of wine, one shot of spirit, or one beer [33 cL]) in the CCHS + CGPS and daily or almost daily intake of alcohol in the UK Biobank. Lipid‐lowering therapy was primarily statins (yes/no) and was self‐reported in all cohorts. Physical inactivity was ≤ 4 hours per week of light physical activity in leisure time in the CCHS + CGPS and ≤ 3 days of week with > 10 minutes of moderate‐intensity physical activity in the UK Biobank. Low education was ≤ 8 years in the CCHS + CGPS and ≤ 7 years in the UK Biobank.

Abbreviations: CCHS, Copenhagen City Heart Study; CGPS, Copenhagen General Population Study; LDL, low‐density lipoprotein; HDL, high‐density lipoprotein.

### 3.1 Association between lipid‐lowering drug targets and lipids

The derived genetic risk scores were associated with lower levels of non‐HDL cholesterol. As a measure of instrument strength, *F* statistics were 872 for the *HMGCR* score, 721 for the *NPC1L1* score, 238 for the *PCSK9* score, 47 for the *ANGPTL4* score, 126 for the *LPL* score, and 1795 for the *CETP* score in CCHS and CGPS; corresponding values were 131, 27, 428, 28, 158, and 94 in the UK Biobank, respectively. *F* statistics for individual genetic variants and association with non‐HDL cholesterol, LDL cholesterol, and triglycerides are provided in Table S2 in supporting information. *F* statistics from GLGC are provided in Table S5 in supporting information. For non‐HDL cholesterol, all *F* statistics for variants used in main analyses were greater than the conventional threshold of 10. Associations with other lipids, lipoproteins, and apolipoproteins are provided in Tables S6 and S7 in supporting information.

### 3.2 Cox regression analyses

**FIGURE 2:**
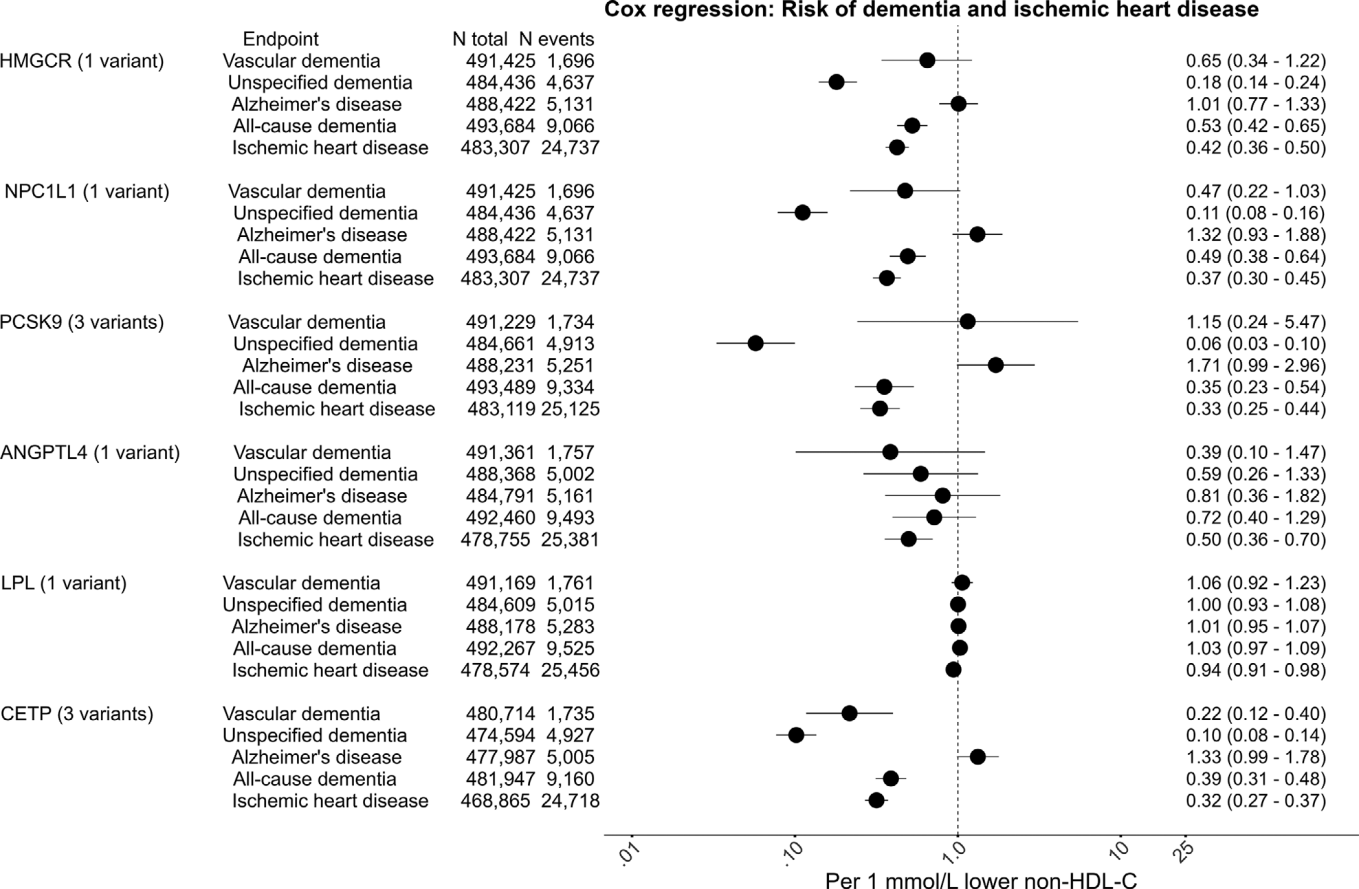
Meta‐analyses of Cox regression results: Risk of dementia and ischemic heart disease in CCHS + CGPS and UK Biobank. The allele scores were calculated based on the allele frequency of the variants in the CCHS + CGPS and UK Biobank. Change in the risk of dementia and ischemic heart disease is per 1 mmol/L (39 mg/dL) genetically lower non‐HDL cholesterol. Adjustment was for age and sex in CCHS + CGPS and for age, sex, and first 10 principal components in UK Biobank. Meta‐analyses for combined studies were performed using the “meta” R package. Heterogeneity tests for meta‐analyses: Q = 0.00–3.29 for *HMGCR*, 0.02–0.64 for *NPC1L1*, 0.82‐3.71 for PCSK9, 0.30–3.86 for *ANGPTL4*, 0.00–8.10 for *LPL*, and 0.16–4.37 for *CETP*. *F* statistics in CCHS + CGPS and the UK Biobank were 872 and 131 for *HMGCR*, 721 and 27 for *NPC1L1*, 238 and 428 for PCSK9, 47 and 28 for *ANGPTL4*, 104 and 158 for *LPL*, and 1795 and 94 for *CETP*, respectively. *ANGPTL4*, angiopoietin like 4; *CETP*, cholesteryl ester transfer protein; CCHS, Copenhagen City Heart Study; CGPS, Copenhagen General Population Study; CI, confidence interval; HDL‐C, high‐density lipoprotein cholesterol; *HMGCR*, β‐hydroxy β‐methylglutaryl‐CoA reductase; HR, hazard ratio; *LPL*, lipoprotein lipase; *NPC1L1*, Nieman pick C1‐like 1; PCSK9, proprotein convertase subtilisin/kexin type 9.

In meta‐analyses of Cox regression results, the hazard ratio (HR) and 95% confidence interval (CI) per 1 mmol/L (39 mg/dL) genetically lower non‐HDL cholesterol by *ANGPTL3* was 0.95 (0.54–1.65) for risk of IHD. The corresponding HR for *APOC3* was 0.96 (0.59–1.58; Figure S1 in supporting information). In meta‐analyses of Cox regression results, the HR and 95% CI per 1 mmol/L (39 mg/dL) genetically lower non‐HDL cholesterol by *HMGCR* was 0.53 (0.42–0.65) for all‐cause dementia and 0.42 (0.36–0.50) for IHD (Figure 2). For *NPC1L1*, the HRs were 0.49 (0.38–0.64) for all‐cause dementia and 0.37 (0.30–0.45) for IHD. For *PCSK9*, the HRs were 0.35 (0.23–0.54) for all‐cause dementia and 0.33 (0.25–0.44) for IHD; for *ANGPTL4*, 0.72 (0.40–1.29) for all‐cause dementia and 0.50 (0.36–0.70) for IHD; for *LPL*, the corresponding HRs were 1.03 (0.97–1.09) for all‐cause dementia and 0.94 (0.91–0.98) for IHD; for *CETP*, 0.39 (0.31–0.48) for all‐cause dementia and 0.32 (0.27–0.37) for IHD. For results for dementia subtypes, see Figure 2, and for cohort‐specific estimates, see Tables S8 and S9 in supporting information. For Cox regression models there was no suspicion of non‐proportionality when viewing plots of the negative natural logarithm of survival versus the natural logarithm of analysis time.

### 3.3 One‐sample Mendelian randomization analyses

**FIGURE 3:**
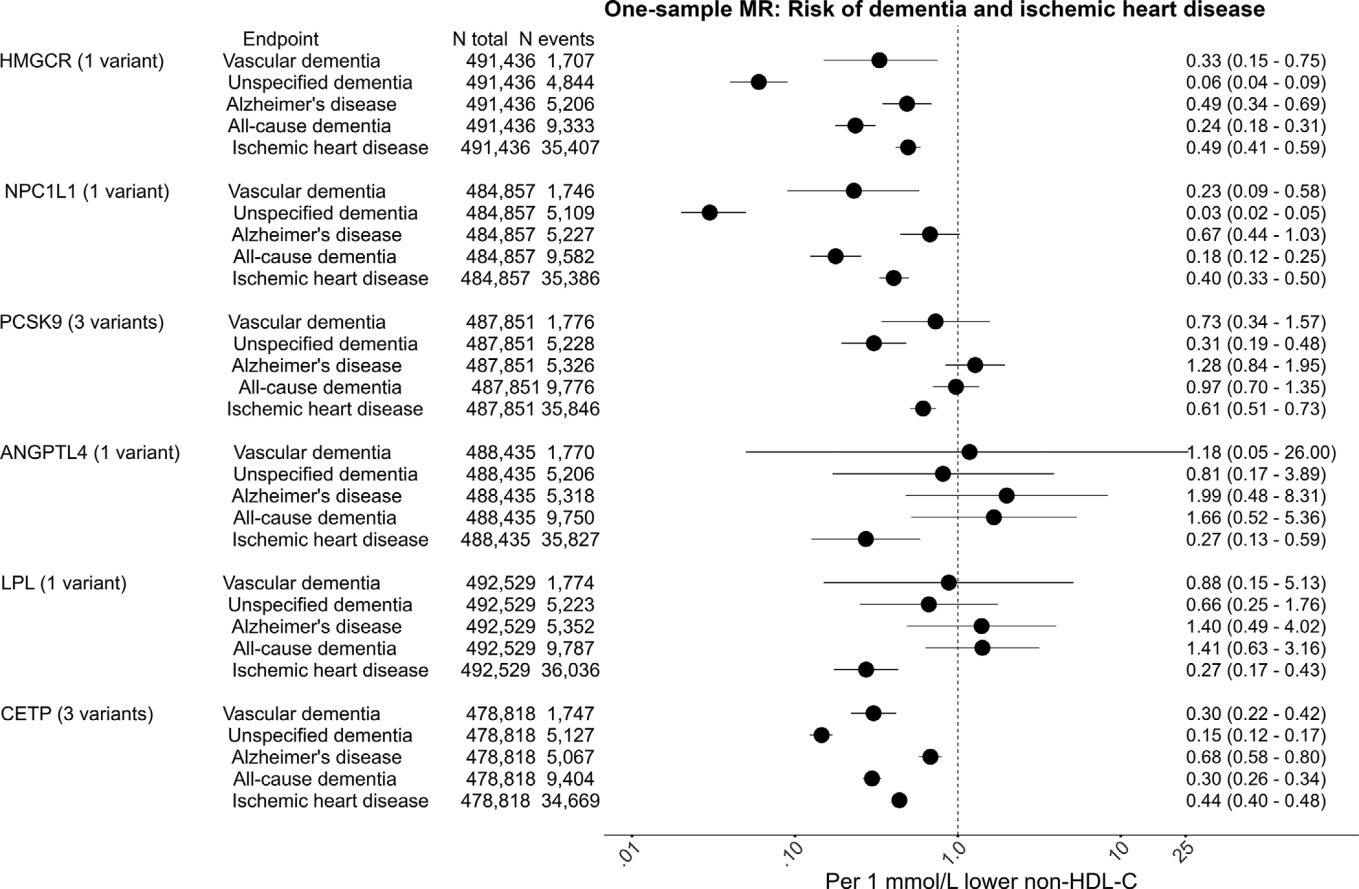
Meta‐analyses of one‐sample Mendelian randomization results: Risk of dementia and ischemic heart disease in CCHS + CGPS and UK Biobank. Individual genetic variants were used as instruments in the CCHS + CGPS and UK Biobank. Change in the risk of dementia and ischemic heart disease is per 1 mmol/L (39 mg/dL) genetically lower non‐HDL cholesterol. The Mendelian randomization estimates were derived using the OneSampleMR package. Meta‐analyses were performed using the meta R package. Heterogeneity tests for meta‐analyses: Q = 0.00–31.60 for *HMGCR*, 0.02–2.04 for *NPC1L1*, 2.1‐47.6 for *PCSK9*, 1.90–4.94 for *ANGPTL4*, 0.02–2.41 for *LPL*, and 0.20–4.92 for *CETP*. *F* statistics in CCHS + CGPS and the UK Biobank were 872 and 131 for *HMGCR*, 721 and 27 for *NPC1L1*, 238 and 428 for PCSK9, 47 and 28 for *ANGPTL4*, 104 and 158 for *LPL*, and mean *F* statistics were 1751 and 93 for *CETP*, respectively. CI, confidence interval; CCHS, Copenhagen City Heart Study; CGPS, Copenhagen General Population Study; *ANGPTL4*, angiopoietin like 4; *CETP*, cholesteryl ester transfer protein; HDL‐C, high‐density lipoprotein cholesterol; *HMGCR*, β‐hydroxy β‐methylglutaryl‐CoA reductase; HR, hazard ratio; *LPL*, lipoprotein lipase; MR, Mendelian randomization; *NPC1L1*, Nieman pick C1‐like 1; OR, odds ratio; PCSK9, proprotein convertase subtilisin/kexin type 9.

In meta‐analyses of Mendelian randomization results, the odds ratio (OR) and 95% CI per 1 mmol/L (39 mg/dL) lower non‐HDL cholesterol by *HMGCR* was 0.24 (0.18–0.31) for all‐cause dementia and 0.49 (0.42–0.59) for IHD (Figure 3). For *NPC1L1* ORs were 0.18 (0.12–0.25) for all‐cause dementia and 0.40 (0.33–0.50) for IHD; for *PCSK9*, 0.97 (0.70–1.35) for all‐cause dementia and 0.61 (0.51–0.73) for IHD; for *ANGPTL4*, 1.66 (0.52–5.36) for all‐cause dementia and 0.27 (0.13–0.59) for IHD; for *LPL*, 1.41 (0.63–3.16) for all‐cause dementia and 0.27 (0.17–0.43) for IHD; and for *CETP*, 0.30 (0.26–0.34) for all‐cause dementia and 0.44 (0.40–0.48) for IHD. For results for dementia subtypes, see Figure 3; for sensitivity analyses, see Table S10 in supporting information; and for cohort‐specific estimates, see Tables S11 and S12 in supporting information.

### 3.4 Two‐sample Mendelian randomization analyses

**FIGURE 4:**
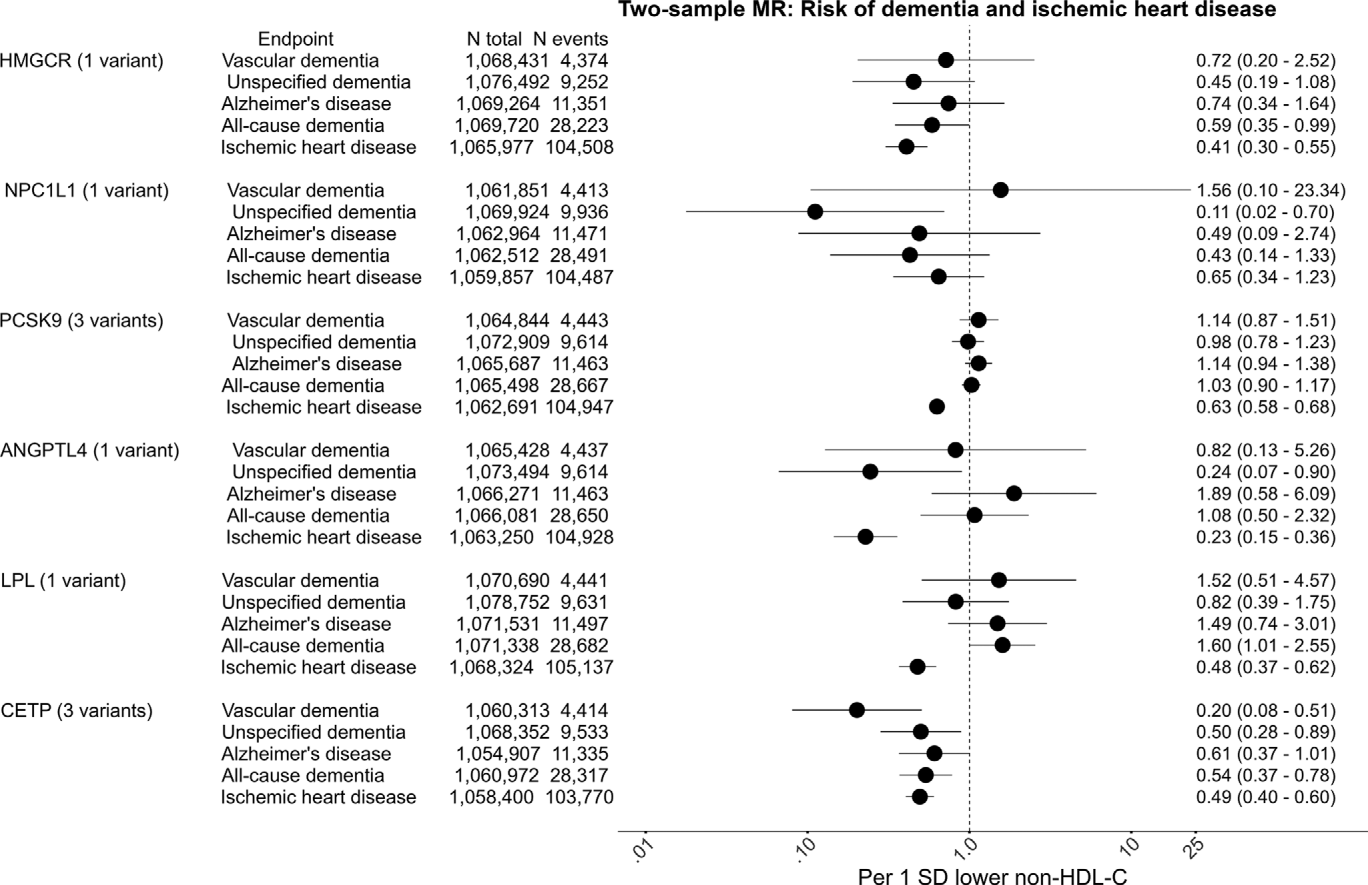
Meta‐analyses of two‐sample Mendelian randomization results: Risk of dementia and ischemic heart disease in CCHS + CGPS, UK Biobank, and FinnGen. Individual genetic variants were used as instruments in the CCHS + CGPS, UK Biobank, FinnGen, and GLGC. Change in the risk of dementia and ischemic heart disease is per 1 SD genetically lower non‐HDL cholesterol. The Mendelian randomization estimates were derived using the TwoSampleMR package. Meta‐analyses were performed using the meta R package. Results are from Wald ratio for drug target genes with one instrument and from weighted median analyses for drug targets with three variants. Heterogeneity tests for meta‐analyses: Q = 0.32–3.83 for *HMGCR*, 0.39–2.05 for *NPC1L1*, 0.26‐77.7 for PCSK9, 1.26–5.56 for *ANGPTL4*, 0.71–1.30 for *LPL*, and 9.19–21.15 for *CETP*. *F* statistics in GLGC were 268 for *HMGCR*, 62 for *NPC1L1*, the mean F statistic was 661 for *PCSK9*, 103 for *ANGPTL4*, 377 for *LPL*, and the mean *F* statistic was 219 for *CETP*, respectively. *ANGPTL4*, angiopoietin like 4; *CETP*, cholesteryl ester transfer protein; CI, confidence interval; *HMGCR*, β‐hydroxy β‐methylglutaryl‐CoA reductase; HR, hazard ratio; *LPL*, lipoprotein lipase; MR, Mendelian randomization; *NPC1L1*, Nieman pick C1‐like 1; OR, odds ratio; PCSK9, proprotein convertase subtilisin/kexin type 9; SD, standard deviation.

In meta‐analyses of two‐sample Mendelian randomization, the OR and 95% CI per 1 standard deviation genetically lower non‐HDL cholesterol by *HMGCR* was 0.59 (0.35–0.99) for all‐cause dementia and 0.41 (0.30–0.55) for IHD using the Wald ratio method (Figure 4). For *NPC1L1*, corresponding ORs were 0.43 (0.14–1.33) for all‐cause dementia and 0.65 (0.34–1.23) for IHD; for *ANGPTL4*, 1.08 (0.50–2.32) for all‐cause dementia and 0.23 (0.15–0.36) for IHD; and for *LPL*, 1.60 (1.01–2.55) for all‐cause dementia and 0.48 (0.37–0.62) for IHD. For *PCSK9*, these ORs were 1.03 (0.90–1.17) for all‐cause dementia and 0.63 (0.58–0.68) for IHD, and for *CETP*, 0.54 (0.37–0.78) for all‐cause dementia and 0.49 (0.40–0.60) for IHD using the weighted median method. For results for dementia subtypes, see Figure 4; for cohort‐specific estimates see Tables S13–S15 in supporting information; and for sensitivity analyses, see Figures S2 and S3 and Tables S16–S18 in supporting information.

### 3.5 Sensitivity analyses

In sensitivity analyses we tested the associations of individual genetic variants with plasma levels of lipids, lipoproteins, and apolipoproteins, including those that were not the main exposure (Tables S6 and S7). In CCHS and CGPS the rs5909 in *HMGCR* was associated with all lipids and lipoproteins investigated except triglycerides and apolipoprotein A1. The rs217434 variant in *NPC1L1* was associated with levels of non‐HDL cholesterol, LDL cholesterol, HDL cholesterol, total cholesterol, and triglycerides, and the *PCSK9* variants were associated with levels of non‐HDL cholesterol, total cholesterol, LDL cholesterol, and apolipoprotein B. The rs116843064 in *ANGPTL4* was associated with all lipids and lipoproteins investigated except lipoprotein (a), and the rs328 variant in *LPL* was associated with all lipids and lipoproteins investigated except total cholesterol. Variants in *CETP* were associated with all lipids and lipoproteins investigated except lipoprotein (a). Results were similar in UK Biobank. For drug targets with more than one genetic instrument used in one‐sample Mendelian randomization, Sargan statistics were calculated. In analyses using a restricted number of variants, there was some evidence of heterogeneity for *CETP* in the CCHS and CGPS, but not in the UK Biobank. In analyses using all variants, there was evidence of pleiotropy in analyses of risk of unspecified dementia (*HMGCR, NPC1L1, LPL*, and *CETP*) and all‐cause dementia (*HMGCR, NPC1L1*, and *CETP*) in CCHS and CGPS but not in UK Biobank. For instruments used in two‐sample Mendelian randomization, tests for pleiotropy and heterogeneity were performed (Tables S16–S18). Results from the TwoSampleMR package “mr_pleiotropy_test” showed Egger intercepts close to 0 (−0.004 to 0.77) with all *P* values > 0.39, indicating little evidence of pleiotropy. There was evidence of heterogeneity in CCHS and CGPS, but very little in UK Biobank or FinnGen. Further, we categorized dementia cases according to whether they carried zero, one, or two copies of the *APOE* ɛ4 allele and performed one‐sample Mendelian randomization analyses. The rationale behind this is to investigate whether the current findings are independent of *APOE* genotype, as *APOE* is the strongest genetic risk factor for dementia. Meta‐analyses of these results showed a reduced risk of all‐cause dementia in individuals with dementia and zero or one *APOE* ɛ4 alleles when non‐HDL cholesterol was lowered by variants *HMGCR, NPC1L1*, and *CETP*. Further, non‐HDL cholesterol lowering by *HMGCR, NPC1L1, LPL*, and *CETP* caused a reduced risk of all‐cause dementia in individuals with dementia and two *APOE* ɛ4 alleles (Figure S4 in supporting information). We also included all variants available in the CCHS and CGPS in one‐sample Mendelian randomization and performed meta‐analyses of results from CCHS and CGPS combined and UK Biobank. Results were similar to those in main analyses (Figure S5 and Tables S19 and S20 in supporting information). When calculating Sargan statistics using all available variants, there was evidence of heterogeneity (Q *P* value < 0.05) in CCHS and CGPS in analyses including all genes and all‐cause dementia and unspecified dementia as endpoints. In UK Biobank there was evidence of heterogeneity (Q *P* value < 0.05) in analyses including *HMGCR* and IHD (Table S21 in supporting information). Finally, we used LDL cholesterol as exposure for *HMGCR, NPC1L1, PCSK9*, and *CETP* and triglycerides for *ANGPTL4* and *LPL* instead of non‐HDL cholesterol in one‐sample Mendelian randomization analyses. Results were directionally consistent with main analyses except for AD, which was null in these analyses (Figures S6 and S7 in supporting information). For reporting of compliance with guidelines for Mendelian randomization studies, see STROBE‐MR checklist.

## 4 DISCUSSION

In this study we investigated the evidence for a causal relationship between non‐HDL cholesterol‐lowering drug targets with vascular dementia, unspecified dementia, AD, and a combination of these endpoints called all‐cause dementia. Our results reflect the effect of lifelong differences in non‐HDL cholesterol on risk of dementia and support that non‐HDL cholesterol lowering through *HMGCR*, *NPC1L1*, and *CETP* reduces the risk of all‐cause dementia; that is, the present data suggest that cholesterol lowering earlier in life likely will reduce the risk of dementia later in life. The nominal effects were larger for vascular dementia and unspecified dementia than for AD. However, except for *CETP* genetic variants, most results for vascular dementia had *P* values > 0.05. This could be due to a lack of power for this endpoint (*n* ≈ 1700 compared to *n* ≈ 5000 for unspecified dementia and AD, respectively). We cannot exclude a causal role of non‐HDL cholesterol lowering through *PCSK9, ANGPTL4*, and *LPL* on risk of dementia. As a positive control, we found all non‐HDL cholesterol‐lowering drug targets to reduce the risk of IHD through all analyses except for *NPC1L1* in two‐sample Mendelian randomization; the latter could be due to weak instrument bias as discussed below.

Accumulating evidence is suggesting that many risk factors for atherosclerotic cardiovascular disease are shared with AD and dementia in general.^2^ The associations between these risk factors and risk of dementia vary according to at which time point in life they have been measured and which type of dementia is the outcome. The strength of the evidence behind these shared associations between cardiovascular risk factors and AD and other types of dementia also varies considerably. Currently, the best evidence is found for high LDL cholesterol in mid‐life and risk of vascular dementia and unspecified dementia; high blood pressure and risk of AD, vascular dementia, and unspecified dementia; and diabetes and risk of AD, vascular dementia, and unspecified dementia.^2^ Risk factors such as diet, smoking, and physical inactivity are less well‐established risk factors.^36^

The proposed biological mechanism underlying the current findings is through the causal effect of elevated non‐HDL cholesterol on the development of atherosclerosis and atherosclerotic cardiovascular disease. This could possibly explain the smaller effect of non‐HDL cholesterol‐lowering drug targets on risk of AD compared to vascular dementia because atherosclerosis is considered to be more important to the pathogenesis of vascular dementia than in AD.^2^ For unspecified dementia there is no current consensus on the pathogenesis.^2^ Because non‐HDL cholesterol comprises cholesterol in all lipoprotein particles except HDL, it includes LDL cholesterol and cholesterol from triglyceride‐rich lipoproteins such as very‐low‐density lipoproteins and intermediate‐density lipoproteins (Figure S8 in supporting information). Of these, LDL cholesterol has most robustly been shown to cause atherosclerotic cardiovascular disease.^37^, ^38^ Importantly, elevated LDL cholesterol is causally affecting not only risk of IHD but also ischemic stroke,^39^, ^40^ secondary to IHD,^41^ and stroke that occurs in the brain is one of the main causes of vascular dementia. While infarctions in the brain are a leading cause of vascular dementia,^42^ infarctions are also likely to be important for the development of unspecified dementia, as many of the dementia cases in this category are suspected to be of vascular origin.^2^, ^4^, ^36^ In the first report of AD, arteriosclerosis was also mentioned as one of the hallmarks of this disease.^43^

In support of our findings for HMGCR, Zhang et al. also found LDL cholesterol lowering by *HMGCR* genetic variants to be associated with reduced risk of vascular dementia in a two‐sample Mendelian randomization study.^44^ However, the authors did not find any evidence of causal effects between other LDL cholesterol‐lowering drug targets (*PCSK9* and *NPC1L1*) and risk of vascular dementia. In one‐sample and two‐sample Mendelian randomization, we did not find evidence of an effect of *PCSK9* variants on risk of all‐cause dementia, but results from Cox regression showed a protective effect. In a study by Benn et al., variants in *HMGCR* were found to be associated with risk of vascular dementia with a HR of 0.44 (0.21–0.89).^45^ Studies investigating the effect of statins (that target HMGCR) on risk of dementia have shown conflicting results. In a large meta‐analysis from 2018, statin use was associated with a 20% reduction in risk of all‐cause dementia, and a 15% reduction in another large meta‐analysis.^46^, ^47^ However, a Cochrane Review from 2016 did not find any effect of statins on risk of dementia.^48^ Recently, the updated recommendations on dementia prevention, intervention, and care included high LDL cholesterol in mid‐life as a modifiable risk factor for dementia prevention.^3^ However, these recommendations are based on meta‐analyses of cohort studies and thus were unable to establish causality.^49^, ^50^ Observationally, triglycerides have been shown to be associated with risk of dementia;^4^ however, current evidence is insufficient to support a causal effect.^44^ In our study, genetic variation in *ANGPTL4* and *LPL* that primarily lowers triglycerides does not affect the risk of vascular dementia or AD. In our two‐sample Mendelian randomization and sensitivity analyses, there is some evidence of an effect on unspecified dementia, dementia in individuals with two *APOE* ɛ4 alleles, and all‐cause dementia for these drug targets. However, these findings are not consistent with findings from our Cox regression or one‐sample Mendelian randomization analyses. When stratifying dementia cases into carriers of zero, one, or two *APOE* ɛ4 alleles, we saw very similar effects of the non‐HDL cholesterol‐lowering drug targets as in the main analyses (Figure S4). Although it has been suggested that *APOE* ɛ4 conveys a certain resistance to extra cardiovascular risk, we did not observe this in our analysis. Finally, we have previously shown that genetic variants in CETP weighted on lower non‐HDL cholesterol were associated with lower risk of vascular dementia but not AD in the CCHS and CGPS combined. However, to our knowledge, unspecified dementia and the composite endpoint of all‐cause dementia have not been previously tested.^11^

Strengths of the current study include using large, prospective cohorts of the general population in Denmark and the UK with individual‐level data and two large consortia with summary‐level data. Both cohorts have relatively long follow‐up periods (CCHS and CGPS, median follow‐up: 12 years [range: 0.1–46 years]; UK Biobank median follow‐up: 13 years [range: 0.1–15 years]), making themsuitable for the study of age‐related diseases such as dementia. Also, by using different cohorts and individual‐level data, summary‐level data, and meta‐analyses of results, we have aimed to provide as robust evidence as possible. Finally, including IHD as a well‐established positive control of the effect of antiatherogenic drug targets confirms the plausibility of our instruments.

Limitations include studying only White Europeans, which might limit the generalizability of our findings to other ethnic groups. Second, dementia diagnoses can be misclassified as they rely on clinical symptoms, and mixed pathology is very common.^2^ Third, because data on principal components were not available in the CCHS and CGPS, we were not able to adjust for these. Fourth, because dementia is an age‐related disease and its prodromal phases can last decades,^51^, ^52^ studies with very long follow‐up times are most ideal for investigating dementia. However, we have included the largest cohorts with information on genetic data and different dementia outcomes and have performed the most well‐powered analyses with the data that is currently available to us. Fifth, the Mendelian randomization approach relies on the gene–environment equivalence principle,^14^ although in practice, there are often differences between the effect of a genetic variant and a proposed intervention.^53^ However, in this study the aim is mainly to establish the presence and direction of the effects of lipid‐lowering drug targets on risk of dementia. Sixth, in Mendelian randomization, pleiotropy is always a risk; however, by selecting few, well‐known variants all within the investigated drug target genes, this risk is minimized, and sensitivity analyses did not imply the presence of pleiotropy.

It is important to acknowledge that this study investigates the effect of genetic targets and not the effect of prescribed medications on dementia risk. A previous study comparing Mendelian randomization studies to prospective cohort studies and randomized clinical trials of the effect of LDL cholesterol lowering on risk of coronary heart disease has found the effect to be largest in Mendelian randomization studies.^37^ This is most likely due to the lifelong effect of genetic variants (median 52 years follow‐up) compared to the relatively short‐term effect in prospective cohort studies (median 12 years follow‐up) and randomized clinical trials (median 5 years follow‐up). It is thus unclear whether cholesterol‐lowering treatment in late life will affect dementia risk, particularly if early phases of dementia have already developed; however, the present data suggest that cholesterol‐lowering earlier in life likely will reduce the risk of dementia later in life. For several shared risk factors between dementia and atherosclerotic cardiovascular disease, including LDL cholesterol and body mass index, there is a reverse epidemiology phenomenon in which high levels of the risk factors in mid‐life are associated with high risk of dementia, while high levels in late life, in contrast, are associated with low risk of dementia. This phenomenon is most likely due to reverse causation: because in the prodromal phases of dementia, loss of initiative and apathy and changes in eating behavior, appetite, and olfactory function all lead to reduced caloric intake and thereby weight loss and reduction in LDL cholesterol and other lipid levels.^54^ Further, it is worth noting that many medications have pleiotropic effects. For example statins have been proposed to lower AD risk through anti‐inflammatory or other mechanisms,^55^ and this would not have been captured in the current study.

In conclusion, we found that genetic lowering (reflecting lifelong reductions) of non‐HDL cholesterol via *HMGCR*, *NPC1L1*, and *CETP* reduces the risk of all‐cause dementia; that is, the present data suggest that cholesterol lowering earlier in life likely will reduce the risk of dementia later in life. This is important because including cholesterol‐lowering drugs in a global dementia prevention strategy could potentially reduce the societal, economic, and personal consequences of this devastating disease.

## AUTHOR CONTRIBUTIONS

All authors contributed to the study conception, design, and data collection. Material preparation and analysis were performed by Liv Tybjærg Nordestgaard. The first draft of the manuscript was written by Liv Tybjærg Nordestgaard with subsequent editing by all authors. All authors read and approved the final manuscript.

## CONFLICT OF INTEREST STATEMENT

B.G.N. reports consultancies/talks for AstraZeneca, Sanofi, Amgen, Amarin, Novartis, Novo Nordisk, Esperion, Abbott, Ultragenyx, USV, Lilly, Arrowhead, and Marea. V.W. reports support from the UK Medical Research Council Integrative Epidemiology Unit (MC_UU_00032/03), grants from Horizon; “EU PROACT: A European proactive adaptive clinical trials network,” NIHR; “Evaluation of COVID‐19 vaccine products and schedules,” NIHR; “Impact and inequalities of winter pressures in primary care: providing the evidence base for mitigation strategies”; and honoraria for presentation at the 2nd International Conference on High‐Quality Healthy Ageing in Beijing. G.D.S. reports grants from the Medical Research Council and scientific advisory board membership for Bristol Myers Squibb, Relation Therapeutics, and Insitro. Other authors have no relevant financial or non‐financial interests to disclose. Author disclosures are available in the supporting information.

## CONSENT STATEMENT

All human subjects provided informed consent.

## Supporting information



Supporting Information

Supporting Information

Supporting Information

## Data Availability

According to Danish law on data sharing, data cannot be made publicly available for the Copenhagen cohorts; however, through reasonable requests to the corresponding author, additional analyses can be conducted in these cohorts. UK Biobank data can be accessed through application. Consortia data can be accessed via https://www.finngen.fi/en/access_results and https://www.lipidgenetics.org/#data‐downloads‐title.
