# Supplementary material for: Cholesterol‐lowering drug targets reduce risk of dementia: Mendelian randomization and meta‐analyses of 1 million individuals
Source: Alzheimers Dement. 2025 Oct 8;21(10):e70638. doi: 10.1002/alz.70638 (PMC12505197; doi:10.1002/alz.70638)
Supplement: Supplementary file 1 — Supporting Information [file ALZ-21-e70638-s002.docx]

**Supplementary Information**

**Cholesterol-lowering drug targets reduce risk of dementia:**

**Mendelian randomization and meta-analyses of 1 million individuals.**

Liv Tybjærg Nordestgaard, Aimee Hanson, Eleanor Sanderson, Emma Anderson, Venexia Walker, Anne Tybjærg-Hansen, George Davey Smith, Børge G. Nordestgaard

Corresponding author: Liv Tybjærg Nordestgaard

Content

[Supplementary Table 1. Genetic variants in lipid lowering drug targets available in CCHS+CGPS. 4](#_Toc192500243)

[Supplementary Table 2. F-stats for non-HDL cholesterol, LDL cholesterol, and triglycerides in CCHS+CGPS and UK Biobank. 5](#_Toc192500244)

[Supplementary Table 3. Diagnoses of diseases according to the International Classification of Disease (ICD) in CCHS+CGPS and UK Biobank. 6](#_Toc192500245)

[Supplementary Table 4. *APOE* genotype distribution for vascular dementia, unspecified dementia, Alzheimer’s disease, and ischemic heart disease cases in the CCHS+CGPS and UK Biobank. 7](#_Toc192500246)

[Supplementary Table 5. Information on effect on non-HDL cholesterol for variants used as instruments in GLGC. 8](#_Toc192500247)

[Supplementary Table 6. Lipid, lipoprotein, and apolipoprotein levels as a function of genotype for variants included in main analyses in the Copenhagen General Population Study and the Copenhagen City Heart Study combined. 9](#_Toc192500248)

[Supplementary Table 7. Lipid, lipoprotein, and apolipoprotein levels as a function of genotype for variants included in main analyses in the UK Biobank 12](#_Toc192500249)

[Supplementary Table 8. Cox regression: Risk of dementia and ischemic heart disease in CCHS+CGPS. 15](#_Toc192500250)

[Supplementary Table 9. Cox regression: Risk of dementia and ischemic heart disease in UK Biobank. 16](#_Toc192500251)

[Supplementary Table 10. Sargan Statistics for genetic instruments with more than one variant used in main analyses. 17](#_Toc192500252)

[Supplementary Table 11. One-sample MR using restricted variants: Risk of dementia and ischemic heart disease in CCHS+CGPS. 18](#_Toc192500253)

[Supplementary Table 12. One-sample MR using restricted number of variants: Risk of dementia and ischemic heart disease in UK Biobank. 19](#_Toc192500254)

[Supplementary Table 13. Two-sample MR using restricted number of variants: Risk of dementia and ischemic heart disease in CCHS+CGPS. 20](#_Toc192500255)

[Supplementary Table 14. Two-sample MR using restricted number of variants: Risk of dementia and ischemic heart disease in UK Biobank. 22](#_Toc192500256)

[Supplementary Table 15. Two-sample MR using restricted number of variants: Risk of dementia and ischemic heart disease in FinnGen. 24](#_Toc192500257)

[Supplementary Table 16. Analyses of pleiotropy in two-sample MR analyses. 25](#_Toc192500258)

[Supplementary Table 17. Analyses of heterogeneity in two-sample MR analyses. 27](#_Toc192500259)

[Supplementary Table 18. Analyses of heterogeneity in two-sample MR analyses. 29](#_Toc192500260)

[Supplementary Table 19. One-sample MR using all available variants: Risk of dementia and ischemic heart disease in CCHS+CGPS. 30](#_Toc192500261)

[Supplementary Table 20. One-sample MR using all available variants: Risk of dementia and ischemic heart disease in the UK Biobank. 32](#_Toc192500262)

[Supplementary Table 21. Sargan Statistics for genetic instruments with more than one variant used in sensitivity analyses including all variants. 33](#_Toc192500263)

[Supplementary Figure 6. Mendelian randomization: Risk of dementia and ischemic heart disease in CCHS+CGPS and UK Biobank. 40](#_Toc192500264)

[Supplementary Figure 7. Mendelian randomization: Risk of dementia and ischemic heart disease in CCHS+CGPS and UK Biobank. 42](#_Toc192500265)

[Supplementary Figure 8. Illustration of plasma lipids, lipoproteins, and apolipoproteins. 44](#_Toc192500266)

[References 45](#_Toc192500267)

#

| **Gene** | **rsid** | **Chr** | **Chr position** | **Major allele** | **Minor allele** | **Consequence** |
| --- | --- | --- | --- | --- | --- | --- |
| ***HMGCR*** | **rs5909**** | 5 | 75360350 | G | A | 3 prime UTR variant |
| *HMGCR* | rs12654264 | 5 | 75352778 | A | T | Intron variant |
| *HMGCR* | rs17238484 | 5 | 75352671 | G | T | Intron variant |
| ***NPC1L1*** | **rs217434**** | 7 | 44513639 | A | G | p.Val1296Val |
| *NPC1L1* | 708272 | 7 | 44541277 | G | T | 5 prime UTR variant |
| ***PCSK9*** | **rs505151**** | 1 | 55063514 | A | G | p.Gly670Glu |
| ***PCSK9*** | **rs562556**** | 1 | 55058564 | A | G | p.Val474Ile |
| ***PCSK9*** | **rs11591147**** | 1 | 55039974 | G | T | p.Arg46His |
| *PCSK9* | rs148195424 | 1 | 55052701 | C | T | p.Arg237Trp |
| *PCSK9* | rs11583680 | 1 | 55039995 | C | T | p.Ala53Gly |
| ***ANGTPL4*** | **rs116843064**** | 19 | 8364439 | G | A | p.Glu40Lys |
| *LPL* | rs118204057 | 8 | 19954222 | G | A | p.Gly215Glu |
| *LPL* | rs1801177 | 8 | 19948197 | G | A | p.Asp36Asn |
| *LPL* | rs268 | 8 | 19956018 | A | G | p.Asn318Ser  p.Asn291Ser |
| ***LPL*** | **rs328**** | 8 | 19962213 | C | G | p.Ser474Ter |
| *CETP* | rs11076176 | 16 | 56973534 | T | G | Intron variant |
| ***CETP*** | **rs1800776**** | 16 | 56961322 | C | A | Intergenic variant |
| *CETP* | rs5883 | 16 | 56973441 | G | C | p.Phe287= |
| ***CETP*** | **rs708272**** | 16 | 56962376 | G | A | Intron variant |
| *CETP* | rs4783961 | 16 | 56960982 | G | A | Intergenic variant |
| ***CETP*** | **rs5880**** | 16 | 56981179 | G | C | Missense variant |
| *CETP* | rs5882 | 16 | 56982180 | A | G | Missense variant |
| *CETP* | rs4783962 | 16 | 56961126 | C | T | Intergenic variant |

# Supplementary Table 1. Genetic variants in lipid lowering drug targets available in CCHS+CGPS.

Genes and selected single nucleotide variants (SNVs) used as instruments for each drug target in the current study. *Forward strand Human (GRCh38.p14). **Variants included in analyses restricted to R^2^ ≤ 0.05.ANGPTL4=angiopoietin like 4; CETP=cholesteryl ester transfer protein; HMGCR= β-hydroxy β-methylglutaryl-CoA reductase; LPL=lipoprotein lipase; NPC1L1=Nieman pick C1-like 1; PCSK9 = Proprotein convertase subtilisin/kexin type 9.

.

| **Gene** | **rsid** | | **F-stats CCHS+CGPS** | | | **F-stats UKB** | | | | | **P-value non-HDL-C**  **GLGC** |
| --- | --- | --- | --- | --- | --- | --- | --- | --- | --- | --- | --- |
|  |  | | **Non-HDL-C** | **LDL-C** | **Triglycerides** | | **Non-HDL-C** | | **LDL-C** | **Triglycerides** |  |
| ***HMGCR*** | **rs5909**** | | **872** | **98** | 5 | **131** | | **143** | | 3 | **3.99*10^-80^** |
| *HMGCR* | rs12654264 | | 105 | 153 | 1 | 598 | | 709 | | 6 | 4.94*10^-324^ |
| *HMGCR* | rs17238484 | | 403 | 75 | 0 | 301 | | 369 | | 0 | 2.72*10^-189^ |
| ***NPC1L1*** | **rs217434**** | | **721** | **33** | 16 | **27** | | **23** | | 3 | **3.16*10^-18^** |
| *NPC1L1* | rs41279633 | | 31 | 39 | 0.02 | 137 | | 148 | | 2 | 7.73*10^-100^ |
| ***PCSK9*** | **rs505151**** | | **38** | **42** | 1 | **366** | | **109** | | 1.7 | 1*10^-124^ |
| ***PCSK9*** | **rs562556**** | | **188** | **28** | 6 | **59** | | **73** | | 1.2 | 3*10^-101^ |
| ***PCSK9*** | **rs11591147**** | | **263** | **340** | 1 | **1,389** | | **1,552** | | 1.5 | 5*10^-324^ |
| *PCSK9* | rs148195424 | | 6 | 14 | 1 | 192 | | 203 | | 1.7 | 8.52*10^-38^ |
| *PCSK9* | rs11583680 | | 9 | 12 | 0.5 | 20 | | 23 | | 0.5 | 3.02*10^-20^ |
| ***ANGTPL4*** | | **rs116843064**** | **47** | 5 | **197** | **28** | | 1 | | **617** | **5.24*10^-33^** |
| *LPL* | rs118204057 | | 5 | 1 | 85 | 2 | | 1 | | 215 | 0.0079 |
| *LPL* | rs1801177 | | 12 | 1 | 67 | 36 | | 6 | | 338 | 1.70*10^-29^ |
| *LPL* | rs268 | | 73 | 7 | 279 | 6 | | 3 | | 25 | 1.43*10^-43^ |
| ***LPL*** | **rs328**** | | **104** | 7 | **414** | **158** | | 13 | | **2306** | **5.41*10^-116^** |
| *CETP* | rs11076176 | | 89 | 91 | 9 | 76 | | 48 | | 63 | 1.07*10^-61^ |
| ***CETP*** | **rs1800776**** | | **2303** | 81 | 34 | **13** | | 9 | | 2 | **6.20*10^-8^** |
| *CETP* | rs5883 | | 11 | 14 | 0 | 3 | | 2 | | 0 | 2.06*10^-7^ |
| ***CETP*** | **Rs708272**** | | **143** | 122 | 27 | **211** | | 159 | | 126 | **3.50*10^-156^** |
| *CETP* | rs4783961 | | 53 | 49 | 11 | 50 | | 39 | | 20 | 1.91*10^-47^ |
| ***CETP*** | **rs5880**** | | **2806** | 174 | 45 | **55** | | 36 | | 54 | **2.43*10^-31^** |
| *CETP* | rs5882 | | 23 | 28 | 2 | 24 | | 16 | | 13 | 4.34*10^-21^ |
| *CETP* | rs4783962 | | 732 | 30 | 23 | 8 | | 7 | | 5 | 8.68*10^-14^ |

Supplementary Table 2. F-stats for non-HDL cholesterol, LDL cholesterol, and triglycerides in CCHS+CGPS and UK Biobank. P-values for association with non-HDL cholesterol in Global Lipids Genetics Consortium (GLGC) from dataset restricted to Europeans from https://cvd.hugeamp.org/. *Variants included in analyses restricted to R^2^ ≤ 0.05. **. ANGPTL4=angiopoietin like 4; CETP=cholesteryl ester transfer protein; HMGCR= β-hydroxy β-methylglutaryl-CoA reductase; LPL=lipoprotein lipase; NPC1L1=Nieman pick C1-like 1; PCSK9 = Proprotein convertase subtilisin/kexin type 9.

| Vascular dementia | Unspecified dementia | Alzheimer’s disease | Ischemic heart disease |
| --- | --- | --- | --- |
| NA | ICD-8 290.09, 290.18 and 290.19 | ICD-8 290 | ICD-8 410 to 414 |
| ICD-9 290.4* | ICD-9 294.2* | ICD-9 331.0* | ICD-9 410 to 414* |
| ICD-10 F01 | ICD-10 F03 | ICD-10 F00  ICD-10 G30 | ICD-10: I20 to I25 |

# Supplementary Table 3. Diagnoses of diseases according to the International Classification of Disease (ICD) in CCHS+CGPS and UK Biobank.

*ICD-9 was never implemented in Denmark.

| CCHS+CGPS | Vascular+unspecified dementia (%)* | Alzheimer’s disease(%) | Ischemic heart disease(%) | Total(%) |
| --- | --- | --- | --- | --- |
| e22 | 8 (0.3) | 14 (0.5) | 77 (0.5) | 755 (0.7) |
| e23 | 262 (9.5) | 194 (6.4) | 1,568 (11.2) | 13578 (12.4) |
| e24 | 92 (3.3) | 89 (3.0) | 384 (2.7) | 3,143 (2.9) |
| e33 | 1,316 (47.5) | 1,207 (40.0) | 7943 (56.6) | 60,680 (55.6) |
| e43 | 917 (33.1) | 1,193 (39.6) | 3,665 (26.1) | 27,847 (25.5) |
| e44 | 175(6.3) | 314 (10.4) | 415 (3.0) | 3,178 (2.9) |
|  | 2,770 | 3,011 | 14,042 | 109,181 |

| UKB | Vascular+unspecified dementia(%) | Alzheimer’s disease(%) | Ischemic heart disease(%) | Total(%) |
| --- | --- | --- | --- | --- |
| e22 | 16 (0.4) | 4 (0.2) | 145 (0.7) | 2,481 (0.6) |
| e23 | 270 (7.6) | 125 (5.4) | 2,336 (10.6) | 47,113 (12.2) |
| e24 | 99 (2.8) | 50 (2.2) | 508 (2.3) | 9,475 (2.5) |
| e33 | 1,460 (41.1) | 743 (32.0) | 12,913 (58.8) | 224,789 (58.6) |
| e34 | 1,340 (37.7) | 1,072 (46.2) | 5,496 (25.0) | 90,571 (23.6) |
| e44 | 371 (10.4) | 326 (14.1) | 568 (2.6) | 9,120 (2.4) |
|  | 3,556 | 2320 | 21,966 | 38,3549 |

# Supplementary Table 4. *APOE* genotype distribution for vascular dementia, unspecified dementia, Alzheimer’s disease, and ischemic heart disease cases in the CCHS+CGPS and UK Biobank.

Distribution of *APOE* genotype for the different subtypes of dementia. *According to Danish law on data protection only fields with more than five individuals can be shown why vascular dementia and unspecified dementia is categorized as one.

| Variant | Gene | chromosome | other allele | effect allele | eaf | beta | se | F statistic |
| --- | --- | --- | --- | --- | --- | --- | --- | --- |
| rs5909 | HMGCR | 5 | G | A | 0.1 | 0.055163 | 0.003373 | 267.5368 |
| rs217434 | NPC1L1 | 7 | A | G | 0.22 | 0.019689 | 0.002502 | 61.94121 |
| rs505151 | PCSK9 | 1 | A | G | 0.0415 | 0.080002 | 0.005186 | 237.96 |
| rs562556 | PCSK9 | 1 | A | G | 0.172 | -0.04564 | 0.002704 | 284.95 |
| rs11591147 | PCSK9 | 1 | G | A | 0.00901 | -0.41286 | 0.010805 | 1460.09 |
| rs116843064 | ANGPTL4 | 19 | G | A | 0.0206 | -0.07352 | 0.007248 | 102.8791 |
| rs328 | LPL | 8 | C | G | 0.0974 | -0.06618 | 0.00341 | 376.7199 |
| rs708272 | CETP | 16 | G | A | 0.438 | -0.04736 | 0.002065 | 526.1889 |
| rs1800776 | CETP | 16 | C | A | 0.0644 | 0.018052 | 0.004113 | 19.26737 |
| rs5880 | CETP | 16 | G | C | 0.049 | 0.050119 | 0.004753 | 111.1779 |

Supplementary Table 5. Information on effect on non-HDL cholesterol for variants used as instruments in GLGC. Eaf = effect allele frequency; se = standard error; ANGPTL4=angiopoietin like 4; CETP=cholesteryl ester transfer protein; HMGCR= β-hydroxy β-methylglutaryl-CoA reductase; LPL=lipoprotein lipase; NPC1L1=Nieman pick C1-like 1; PCSK9 = Proprotein convertase subtilisin/kexin type 9.

.

# Supplementary Table 6. Lipid, lipoprotein, and apolipoprotein levels as a function of genotype for variants included in main analyses in the Copenhagen General Population Study and the Copenhagen City Heart Study combined.

|  | ***NPC1L1*** | **rs217434** |  | **P** | ***HMGCR*** | **rs5909** |  | **P** |
| --- | --- | --- | --- | --- | --- | --- | --- | --- |
|  | **GG(8,146)** | **AG(34,491)** | **AA(58,648)** |  | **AA(3,904)** | **AG(18,8206)** | **GG(85,134)** |  |
| **Non-HDL cholesterol, mmol/L** | 4.40(1.25) | 4.05(1.14) | 3.98(1.11) | <0.0001 | 4.63(1.22) | 4.02(1.13) | 3.95(1.10) | <0.0001 |
| **HDL cholesterol, mmol/L** | 1.45(0.51) | 1.60(0.52) | 1.62(0.52) | <0.0001 | 1.34(0.46) | 1.61(0.52) | 1.62(0.52) | <0.0001 |
| **Total cholesterol, mmol/L** | 5.86(1.17) | 5.65(1.10) | 5.60(1.08) | <0.0001 | 5.98(1.16) | 5.63(1.09) | 5.57(1.08) | <0.0001 |
| **LDL cholesterol, mmol/L** | 3.32(1.01) | 3.26(0.96) | 3.24(0.95) | <0.0001 | 3.45(1.09 | 3.27(0.96) | 3.22(0.95) | <0.0001 |
| **Triglycerides, mmol/L** | 1.42(1.0-2.09) | 1.39(0.97-2.05) | 1.39(0.96-2.05) | <0.0001 | 1.37(1.0-2.01) | 1.38(0.96-2.05) | 1.38(0.96-2.04) | 0.28 |
| **Apolipoprotein A1, mg/dL** | 1.62(0.31) | 1.62(0.30) | 1.62(0.30) | 0.84 | 1.63(0.30) | 1.63(0.30) | 1.63(0.30) | 0.80 |
| **Apolipoprotein B, mg/dL** | 1.11(0.34) | 1.11(0.34) | 1.11(0.33) | 0.19 | 1.12(0.33) | 1.10(0.34) | 1.10(0.33) | <0.0001 |
| **Lipoprotein(a), mg/dL** | 11.9(5.89-32.33) | 11.53(5.69-30.32) | 11.62(5.77-31.28) | 0.37 | 10.07(4.99-26.7) | 11.7(5.75-32.3) | 11.2(5.49-30.2) | 0.002 |
|  | ***ANGPTL4***  **GG(99132)** | **rs116843064**  **GA(5,661)** | **AA(70)** | **P** | ***LPL***  **CC(88,840)** | **rs328**  **CG(20,111)** | **GG(1,174)** | **P** |
| **Non-HDL cholesterol, mmol/L** | 4.04(1.14) | 3.95(1.14) | 3.34(1.00) | <0.0001 | 4.04(1.14) | 3.96(1.12) | 3.88(1.05) | <0.0001 |
| **HDL cholesterol, mmol/L** | 1.59(0.52) | 1.69(0.53) | 1.89(0.47) | <0.0001 | 1.58(0.51) | 1.65(0.53) | 1.73(0.52) | <0.0001 |
| **Total cholesterol, mmol/L** | 5.63(1.09) | 5.64(1.09) | 5.22(1.01) | 0.010 | 5.62(1.10) | 5.61(1.09) | 5.61(1.04) | 0.71 |
| **LDL cholesterol, mmol/L** | 3.25(0.96) | 3.23(0.96) | 2.93(0.80) | 0.003 | 3.25(0.96) | 3.23(0.96) | 3.22(0.92) | 0.05 |
| **Triglycerides, mmol/L** | 1.70(1.15) | 1.49(1.03) | 1.07(0.51) | <0.0001 | 1.72(1.18) | 1.55(1.01) | 1.41(0.82) | <0.0001 |
| **Apolipoprotein A1, mg/dL** | 1.62(0.30) | 1.65(0.31) | 1.67(0.33) | <0.0001 | 1.62(0.30) | 1.64(0.31) | 1.67(0.31) | <0.0001 |
| **Apolipoprotein B, mg/dL** | 1.11(0.34) | 1.06(0.31) | 0.89(0.26) | <0.0001 | 1.11(0.34) | 1.07(0.32) | 1.04(0.29) | <0.0001 |
| **Lipoprotein(a), mg/dL** | 11.51(5.7-30.72) | 11.81(5.73-31.65) | 10.0(3.96-24.18) | 0.40 | 11.33(5.59-30.38) | 11.59(5.7-31.71) | 13.48(5.98-36.64) | 0.04 |

|  | | ***CETP***  **GG(31,413)** | **rs708272**  **GA(49,520)** | **AA(18,968)** | **P** | ***CETP***  **AA(5,949)** | **rs1800776**  **AC(12,490)** | **CC(83,971)** | **P** |  |
| --- | --- | --- | --- | --- | --- | --- | --- | --- | --- | --- |
|  | |  |  |  |  |  |  |  |  |  |
| **Non-HDL cholesterol, mmol/L** | | 4.09(1.15) | 4.03(1.13) | 3.96(1.14) | <0.0001 | 4.85(1.22) | 4.05(1.14) | 3.98(1.11) | <0.0001 |  |
| **HDL cholesterol, mmol/L** | | 1.51(0.49) | 1.61(0.51) | 1.73(0.55) | <0.0001 | 1.25(0.41) | 1.57(0.50) | 1.63(0.52) | <0.0001 |  |
| **Total cholesterol, mmol/L** | | 5.60(1.11) | 5.64(1.09) | 5.69(1.08) | <0.0001 | 6.12(1.16) | 5.62(1.10) | 5.61(1.08) | <0.0001 |  |
| **LDL cholesterol, mmol/L** | | 3.29(0.96) | 3.25(0.96) | 3.18(0.95) | <0.0001 | 3.59(1.16) | 3.26(0.95) | 3.24(0.96) | <0.0001 |  |
| **Triglycerides, mmol/L** | | 1.71(1.19) | 1.69(1.13) | 1.66(1.12) | <0.0001 | 1.44(1.04-2.11) | 1.39(0.97-2.06) | 1.39(0.96-2.05) | <0.0001 |  |
| **Apolipoprotein A1, mg/dL** | | 1.58(0.29) | 1.62(0.30) | 1.68(0.31) | <0.0001 | 1.59(0.28) | 1.61(0.30) | 1.62(0.30) | <0.0001 |  |
| **Apolipoprotein B, mg/dL** | | 1.12(0.34) | 1.11(0.33) | 1.09(0.33) | <0.0001 | 1.15(0.34) | 1.11(0.34) | 1.11(0.34) | 0.03 |  |
| **Lipoprotein(a), mg/dL** | | 11.98(5.93-31.1) | 11.5(5.69-30.82) | 11.11(5.6-30.6) | 0.002 | 11.94(5.76-30.26)( | 11.31(5.69-29.1) | 11.6(5.74-31.1) | 0.33 |  |
|  | | ***CETP***  **GG(84,428)** | **rs5880**  **GC(9,832)** | **CC(5,951)** | **P** | ***PCSK9***  **GG(127)** | **rs505151**  **GA(7,236)** | **AA(92,887)** | P |  |
| **Non-HDL cholesterol, mmol/L** | | 3.97(1.11) | 4.10(1.14) | 4.86(1.12) | <0.0001 | 4.14(1.24) | 4.09(1.14) | 4.00(1.12) | <0.0001 |  |
| **HDL cholesterol, mmol/L** | | 1.64(0.52) | 1.48(0.48) | 1.24(0.40) | <0.0001 | 1.61(0.47) | 1.60(0.51) | 1.61(0.52) | 0.13 |  |
| **Total cholesterol, mmol/L** | | 5.61(1.08) | 5.59(1.10) | 6.12(1.18) | <0.0001 | 5.75(1.12) | 5.69(1.09) | 5.52(1.09) | <0.0001 |  |
| **LDL cholesterol, mmol/L** | | 3.23(0.95) | 3.31(0.97) | 3.58(1.13) | <0.0001 | 3.36(1.05) | 3.32(0.98) | 3.24(0.96) | <0.0001 |  |
| **Triglycerides, mmol/L** | | 1.68(1.12) | 1.71(1.20) | 1.79(1.51) | <0.0001 | 1.37(0.94-2.2) | 1.39(1.0-2.1) | 1.38(1.0-2.1) | 0.55 |  |
| **Apolipoprotein A1, mg/dL** | | 1.63(0.30) | 1.56(0.28) | 1.49(0.26) | <0.0001 | 1.61(0.28) | 1.61(0.30) | 1.62(0.30) | 0.42 |  |
| **Apolipoprotein B, mg/dL** | | 1.11(0.34) | 1.12(0.34) | 1.13(0.34) | <0.0001 | 1.14(0.38) | 1.12(0.34) | 1.11(0.33) | <0.0001 |  |
| **Lipoprotein(a), mg/dL** | | 11.5(5.7-30.7) | 11.7(6.0-31.7) | 11.6(6.9-32.0) | 0.32 | 13(7-37) | 12(6-33) | 12(6-31) | 0.15 |  |
|  |  | | | | | | | | | |

|  |  | |  | |  | |  | |  |  |
| --- | --- | --- | --- | --- | --- | --- | --- | --- | --- | --- |
|  | | ***PCSK9***  **AA(70,280)** | | **rs562556**  **GA(26,464)** | | **GG(2,543)** | | **P** | |  |
| **Non-HDL cholesterol, mmol/L** | | 4.03(1.13) | | 3.98(1.11) | | 3.96(1.11) | | <0.0001 | |  |
| **HDL cholesterol, mmol/L** | | 1.61(0.52) | | 1.61(0.52) | | 1.61(0.52) | | 0.44 | |  |
| **Total cholesterol, mmol/L** | | 5.63(1.09) | | 5.59(1.08) | | 5.58(1.10) | | <0.0001 | |  |
| **LDL cholesterol, mmol/L** | | 3.26(0.96) | | 3.21(0.95) | | 3.19(0.94) | | <0.0001 | |  |
| **Triglycerides, mmol/L** | | 1.38(1.0-2.1) | | 1.38(1.0-2.1) | | 1.4(1.0-2.1) | | 0.29 | |  |
| **Apolipoprotein A1, mg/dL** | | 1.62(0.30) | | 1.62(0.30) | | 1.62(0.31) | | 0.68 | |  |
| **Apolipoprotein B, mg/dL** | | 1.11(0.34) | | 1.10(0.33) | | 1.10(0.33) | | <0.0001 | |  |
| **Lipoprotein(a), mg/dL** | | 12(6-31) | | 12(6-31) | | 11(5-30) | | 0.52 | |  |
|  | | ***PCSK9***  **GG(101,333)** | | **Rs11591147**  **GT(2,710)** | | **TT(18)** | | **P** | |  |
| **Non-HDL cholesterol, mmol/L** | | 4.05(1.14) | | 3.69(1.11) | | 3.19(0.84) | | <0.0001 | |  |
| **HDL cholesterol, mmol/L** | | 1.6080.52) | | 1.61(0.52) | | 1.86(0.80) | | 0.10 | |  |
| **Total cholesterol, mmol/L** | | 5.65(1.09) | | 5.31(1.09) | | 5.05(0.94) | | <0.0001 | |  |
| **LDL cholesterol, mmol/L** | | 3.25(1.0) | | 2.91(0.90) | | 2.40(0.84) | | <0.0001 | |  |
| **Triglycerides, mmol/L** | | 1.39(1.0-2.1) | | 1.41(1.0-2.1) | | 1.25(1.1-2.1) | | 0.93 | |  |
| **Apolipoprotein A1, mg/dL** | | 1.62(0.30) | | 1.63(0.31) | | 1.83(0.42) | | 0.008 | |  |
| **Apolipoprotein B, mg/dL** | | 1.11(0.34) | | 1.04(0.34) | | 0.87(0.20) | | <0.0001 | |  |
| **Lipoprotein(a), mg/dL** | | 12(6-31) | | 11(5-34) | | 10(5-42) | | 0.46 | |  |
| Values are mean (SD) for normally distributed variables (non-HDL cholesterol, HDL cholesterol, total cholesterol, LDL cholesterol, apolipoprotein A1, and apolipoprotein B). Values are median (range) for values that are not normally distributed (triglycerides and lipoprotein(a)). *P* values reflect the Kruskal-Wallis test. To convert cholesterol to mg/dL, divide by 0.0259; to convert triglycerides to mg/dL, divide by 0.0113. HDL = high-density lipoprotein; LDL=low-density lipoprotein. ANGPTL4=angiopoietin like 4; CETP=cholesteryl ester transfer protein; HMGCR= β-hydroxy β-methylglutaryl-CoA reductase; HR=hazard ratio; LPL=lipoprotein lipase; NPC1L1=Nieman pick C1-like 1; P = p-value; PCSK9 = Proprotein convertase subtilisin/kexin type 9. | | | | | | | | | | |

# Supplementary Table 7. Lipid, lipoprotein, and apolipoprotein levels as a function of genotype for variants included in main analyses in the UK Biobank

|  | ***NPC1L1*** | **rs217434** |  | **P** | ***HMGCR*** | **rs5909** |  | **P** |
| --- | --- | --- | --- | --- | --- | --- | --- | --- |
|  | **GG(15,739)** | **AG(124,274)** | **AA(243,559)** |  | **AA(3,401)** | **AG(65,339)** | **GG(314,832)** |  |
| **Non-HDL cholesterol, mmol/L** | 4.29(1.08) | 4.27(1.08) | 4.25(1.07) | <0.0001 | 4.36(1.10) | 4.30(1.08) | 4.25(1.08) | <0.0001 |
| **HDL cholesterol, mmol/L** | 1.45(0.38) | 1.45(0.38) | 1.45(0.38) | 0.69 | 1.46(0.38) | 1.45(0.38) | 1.45(0.38) | 0.25 |
| **Total cholesterol, mmol/L** | 5.74(1.15) | 5.72(1.15) | 5.71(1.14) | 0.0002 | 5.82(1.17) | 5.75(1.15) | 5.70(1.14) | <0.0001 |
| **LDL cholesterol, mmol/L** | 3.59(0.87) | 3.58(0.87) | 3.57(0.87) | <0.0001 | 3.66(0.89) | 3.60(0.88) | 3.56(0.87) | <0.0001 |
| **Triglycerides, mmol/L** | 1.50(1.06-2.17) | 1.49(1.06-2.16) | 1.49(1.05-2.15) | 0.03 | 1.50(1.07-2.18) | 1.49(1.05-2.16) | 1.49(1.05-2.16) | 0.32 |
| **Apolipoprotein A1, mg/dL** | 1.54(0.27) | 1.54(0.27) | 1.54(0.27) |  | 1.54(0.27) | 1.54(0.27) | 1.54(0.27) | 0.74 |
| **Apolipoprotein B, md/dL** | 1.04(0.24) | 1.04(0.24) | 1.03(0.24) | 0.0003 | 1.06(0.24) | 1.04(0.24) | 1.03(0.24) | <0.0001 |
| **Lipoprotein(a), nmol/L** | 20.3(9.4-60.1) | 20.6(9.4-60.8) | 20.2(9.2-59.7) | 0.09 | 21.85(9.8-63.0) | 20.4(9.4-60.1) | 20.2(9.37-60) | 0.16 |
|  | ***ANGPTL4***  **GG(368,866)** | **rs116843064**  **GA(14,553)** | **AA(153)** | **P** | ***LPL***  **CC(88,840)** | **rs328**  **CG(20,111)** | **GG(1,174)** | **P** |
| **Non-HDL cholesterol, mmol/L** | 4.26(1.08) | 4.21(1.05) | 4.00(0.95) | <0.0001 | 4.27(1.08) | 4.22(1.06) | 4.16(1.05) | <0.0001 |
| **HDL cholesterol, mmol/L** | 1.45(0.38) | 1.52(0.39) | 1.66(0.42) | <0.0001 | 1.44(0.38) | 1.50(0.39) | 1.55(0.39) | <0.0001 |
| **Total cholesterol, mmol/L** | 5.71(1.14) | 5.74(1.11) | 5.67(1.00) | 0.008 | 5.71(1.15) | 5.72(1.12) | 5.71(1.11) | 0.02 |
| **LDL cholesterol, mmol/L** | 3.57(0.87) | 3.57(0.85) | 3.46(0.78) | 0.29 | 3.57(0.87) | 3.56(0.86) | 3.54(0.85) | 0.002 |
| **Triglycerides, mmol/L** | 1.50(1.06-2.17) | 1.34(0.95-1.92) | 0.97(0.75-1.40) | <0.0001 | 1.52(1.07-2.20) | 1.37(0.98-1.97) | 1.27(0.92-1.79) | <0.0001 |
| **Apolipoprotein A1, mg/dL** | 1.54(0.27) | 1.57(0.27) | 1.61(0.25) | <0.0001 | 1.53(0.27) | 1.57(0.27) | 1.59(0.27) | <0.0001 |
| **Apolipoprotein B, mg/dL** | 1.03(0.24) | 1.03(0.23) | 1.00(21) | 0.01 | 1.04(0.24) | 1.03(0.23) | 1.02(0.23) | <0.0001 |
| **Lipoprotein(a), mg/dL** | 20.3(9.4-60) | 20.5(9.32-60.1) | 25.1(10.2-107) | 0.06 | 20.2(9.3-60) | 20.8(9.5-59.8) | 22.6(10.3-63.2) | <0.0001 |

.

|  | ***CETP***  **GG(123,066)** | **rs708272**  **GA(188,483)** | **AA(72,023)** | **P** | ***CETP***  **AA(2,262)** | **rs1800776**  **AC(54,840)** | **CC(326,470)** | **P** |
| --- | --- | --- | --- | --- | --- | --- | --- | --- |
| **Non-HDL cholesterol, mmol/L** | 4.28(1.08) | 4.26(1.08) | 4.21(1.07) | <0.0001 | 4.30(1.09) | 4.27(1.08) | 4.26(1.08) | 0.002 |
| **HDL cholesterol, mmol/L** | 1.39(0.36) | 1.46(0.38) | 1.54(0.41) | <0.0001 | 1.41(0.37) | 1.43(0.37) | 1.46(0.38) | <0.0001 |
| **Total cholesterol, mmol/L** | 5.67(1.15) | 5.72(1.14) | 5.75(1.13) | <0.0001 | 5.70(1.15) | 5.70(1.15) | 5.71(1.14) | 0.11 |
| **LDL cholesterol, mmol/L** | 3.59(0.88) | 3.57(0.87) | 3.53(0.86) | <0.0001 | 3.60(0.87) | 3.58(0.87) | 3.57(0.89) | 0.01 |
| **Triglycerides, mmol/L** | 1.51(1.06-2.19) | 1.49(1.05-2.15) | 1.46(1.03-2.11) | <0.0001 | 1.51(1.04-2.13) | 1.50(1.06-2.17) | 1.49(1.05-2.15) | 0.02 |
| **Apolipoprotein A1, mg/dL** | 1.51(0.26) | 1.55(0.27) | 1.59(0.28) | <0.0001 | 1.51(0.27) | 1.53(0.27) | 1.54(0.27) | <0.0001 |
| **Apolipoprotein B, mg/dL** | 1.04(0.24) | 1.03(0.24) | 1.02(0.23) | <0.0001 | 1.04(0.24) | 1.04(0.24) | 1.03(0.24) | 0.002 |
| **Lipoprotein(a), mg/dL** | 20.8(9.53-60.1) | 20.3(9.4-60.2) | 19.5(9.1-59.4) | <0.0001 | 21.2(9.6-66.9) | 20.5(9.5-60) | 20.2(9.3-60.0) | 0.05 |
|  | ***CETP***  **CC(1,151)** | **rs5880**  **GC(39,698)** | **TT(342,723)** | **P** | ***PCSK9***  **GG(440)** | **rs505151**  **GA(23,672)** | **AA(359,460)** | **P** |
| **Non-HDL cholesterol, mmol/L** | 4.25(1.07) | 4.30(1.08) | 4.25(1.08) | <0.0001 | 4.38(1.05) | 4.33(1.09) | 4.26(1.08) | <0.0001 |
| **HDL cholesterol, mmol/L** | 1.29(0.33) | 1.37(0.36) | 1.46(0.38) | <0.0001 | 1.48(0.37) | 1.45(0.38) | 1.46(0.38) | 0.33 |
| **Total cholesterol, mmol/L** | 5.54(1.14) | 5.67(1.15) | 5.72(1.15) | <0.0001 | 5.85(1.11) | 5.79(1.15) | 5.72(1.14) | <0.0001 |
| **LDL cholesterol, mmol/L** | 3.55(0.88) | 3.60(0.88) | 3.57(0.87) | <0.0001 | 3.67(0.86) | 3.63(0.88) | 3.57(0.87) | <0.0001 |
| **Triglycerides, mmol/L** | 1.54(1.06-2.20) | 1.52(1.07-2.20) | 1.49(1.05-2.15) | <0.0001 | 1.51(1.03-2.3) | 1.49(1.05-2.2) | 1.50(1.05-2.2) | 0.41 |
| **Apolipoprotein A1, mg/dL** | 1.45(0.25) | 1.49(0.26) | 1.55(0.27) | <0.0001 | 1.55(0.27) | 1.54(0.27) | 1.54(0.27) | 0.08 |
| **Apolipoprotein B, mg/dL** | 1.04(0.24) | 1.05(0.24) | 1.03(0.24) | <0.0001 | 1.05(0.23) | 1.05(0.24) | 1.03(0.24) | <0.0001 |
| **Lipoprotein(a), mg/dL** | 23.1(9.7-60.6) | 21.09(0.6-61.1) | 20.2(9,33-59.9) | 0.0003 | 17.8(9.2-57.0) | 20.5(9.5-60.2) | 20.3(9.4-60.1) | 0.66 |
|  | | | | | | | | |

|  | | | ***PCSK9***  **AA(257,986)** | **rs562556**  **GA(113,189)** | **GG(12,397)** | **P** |  |
| --- | --- | --- | --- | --- | --- | --- | --- |
| **Non-HDL cholesterol, mmol/L** | | | 4.28(1.08) | 4.25(1.08) | 4.22(1.08) | <0.0001 |  |
| **HDL cholesterol, mmol/L** | | | 1.46(0.38) | 1.45(0.38) | 1.46(0.38) | 0.09 |  |
| **Total cholesterol, mmol/L** | | | 5.73(1.14) | 5.71(1.14) | 5.68(1.13) | <0.0001 |  |
| **LDL cholesterol, mmol/L** | | | 3.58(0.87) | 3.56(0.87) | 3.54(0.87) | <0.0001 |  |
| **Triglycerides, mmol/L** | | | 1.49(1.05-2.2) | 1.50(1.05-2.2) | 1.52(1.06-2.2) | 0.14 |  |
| **Apolipoprotein A1, mg/dL** | | | 1.54(0.27) | 1.54(0.27) | 1.55(0.27) | 0.08 |  |
| **Apolipoprotein B, mg/dL** | | | 1.04(0.24) | 1.03(0.24) | 1.02(0.24) | <0.0001 |  |
| **Lipoprotein(a), mg/dL** | | | 20.4(9.4-60.2) | 20.2(9.4-60.2) | 19.5(9.3-58.8) | 0.15 |  |
|  | | | ***PCSK9***  **GG(370,257)** | **Rs11591147**  **GT(13,188)** | **TT(127)** | **P** |  |
| **Non-HDL cholesterol, mmol/L** | | | 4.28(1.08) | 3.93(1.00) | 3.38(1.03) | <0.0001 |  |
| **HDL cholesterol, mmol/L** | | | 1.46(0.38) | 1.45(0.39) | 1.50(0.41) | 0.29 |  |
| **Total cholesterol, mmol/L** | | | 5.74(1.14) | 5.38(1.06) | 4.88(1.09) | <0.0001 |  |
| **LDL cholesterol, mmol/L** | | | 3.59(0.87) | 3.29(0.80) | 2.82(0.81) | <0.0001 |  |
| **Triglycerides, mmol/L** | | | 1.49(1.05-2.2) | 1.50(1.0-2.2) | 1.49(0.89-2.2) | 0.84 |  |
| **Apolipoprotein A1, mg/dL** | | | 1.54(0.27) | 1.56(0.27) | 1.60(0.28) | <0.0001 |  |
| **Apolipoprotein B, mg/dL** | | | 1.04(0.24) | 0.96(0.22) | 0.84(0.22) | <0.0001 |  |
| **Lipoprotein(a), mg/dL** | | | 20.4(9.4-60.2) | 18.9(9.02-58.68) | 15.7(9.5-49.78) | 0.001 |  |
|  | Values are mean (SD) for normally distributed variables (non-HDL cholesterol, HDL cholesterol, total cholesterol, LDL cholesterol, apolipoprotein A1, and apolipoprotein B). Values are median (range) for values that are not normally distributed (triglycerides and lipoprotein(a)). *P* values reflect the Kruskal-Wallis test. To convert cholesterol to mg/dL, divide by 0.0259; to convert triglycerides to mg/dL, divide by 0.0113. HDL = high-density lipoprotein; LDL=low-density lipoprotein. ANGPTL4=angiopoietin like 4; CETP=cholesteryl ester transfer protein; HMGCR= β-hydroxy β-methylglutaryl-CoA reductase; HR=hazard ratio; LPL=lipoprotein lipase; NPC1L1=Nieman pick C1-like 1; P = p-value; PCSK9 = Proprotein convertase subtilisin/kexin type 9. | | | | | | |
|  | |  | | | | | |

| **exposure** | **outcome** | **b** | **se** | **HR** | **CI low** | **CI high** |
| --- | --- | --- | --- | --- | --- | --- |
| ANGPTL4 | AD | 0.71 | 0.73 | 2.033991 | 0.486363 | 8.5062399 |
| ANGPTL4 | AllD | 0.64 | 0.58 | 1.896481 | 0.608475 | 5.9109112 |
| ANGPTL4 | IHD | -1.485 | 0.48 | 0.226502 | 0.088407 | 0.5803058 |
| ANGPTL4 | UD | -0.12 | 0.85 | 0.88692 | 0.167629 | 4.6926619 |
| ANGPTL4 | VD | 1.07 | 1.68 | 2.915379 | 0.108305 | 78.476561 |
| CETP | AD | 0.32224 | 1.52E-01 | 1.380219 | 1.024925 | 1.8586767 |
| CETP | AllD | -0.9402 | 0.1111167 | 0.390554 | 0.314121 | 0.4855851 |
| CETP | IHD | -1.17655 | 0.0840703 | 0.308342 | 0.261499 | 0.3635752 |
| CETP | UD | -2.31101 | 0.1459044 | 0.099161 | 0.074498 | 0.1319889 |
| CETP | VD | -1.58412 | 0.3180257 | 0.205128 | 0.109981 | 0.3825903 |
| HMGCR | AD | 0.04 | 0.14 | 1.040811 | 0.791045 | 1.3694374 |
| HMGCR | AllD | -0.62 | 0.11 | 0.537944 | 0.433614 | 0.6673771 |
| HMGCR | IHD | -0.86 | 0.084 | 0.423162 | 0.358926 | 0.4988948 |
| HMGCR | UD | -1.686 | 0.147 | 0.185259 | 0.138883 | 0.2471203 |
| HMGCR | VD | -0.39 | 0.33 | 0.677057 | 0.354588 | 1.2927865 |
| LPL | AD | 0.01 | 0.03 | 1.01005 | 0.952372 | 1.0712219 |
| LPL | AllD | 0.03 | 0.03 | 1.030455 | 0.971611 | 1.0928621 |
| LPL | IHD | -0.05 | 0.02 | 0.951229 | 0.914663 | 0.9892581 |
| LPL | UD | 0.0006 | 0.04 | 1.0006 | 0.925149 | 1.0822043 |
| LPL | VD | 0.065 | 0.078 | 1.067159 | 0.915871 | 1.2434378 |
| NPC1L1 | AD | 0.27 | 0.18 | 1.309964 | 0.920535 | 1.8641403 |
| NPC1L1 | AllD | -0.71 | 0.13 | 0.491644 | 0.381059 | 0.6343211 |
| NPC1L1 | IHD | -1.01 | 0.1008 | 0.364219 | 0.298923 | 0.4437775 |
| NPC1L1 | UD | -2.19 | 0.18 | 0.111917 | 0.078646 | 0.1592627 |
| NPC1L1 | VD | -0.75 | 0.3989 | 0.472367 | 0.216137 | 1.0323564 |
| PCSK9 | AD | 0.76 | 0.32 | 0.200168 | 0.1379312 | 0.290486 |
| PCSK9 | AllD | -1.44 | 0.25 | 0.236928 | 0.1451482 | 0.386741 |
| PCSK9 | IHD | -1.6086 | 0.19 | 0.200168 | 0.1379312 | 0.290486 |
| PCSK9 | UD | -3.75 | 0.33 | 0.023518 | 0.0123167 | 0.044905 |
| PCSK9 | VD | -2.56 | 72 | 0.077305 | 3.986E-63 | 1.5E+60 |

Supplementary Table 8. Cox regression: Risk of dementia and ischemic heart disease in CCHS+CGPS. Betas and standard errors (se) are from Cox regression models. The allele scores were calculated based on the allele frequency of the variants in the CCHS+CGPS. Change in the risk of dementia and ischemic heart disease is per 1 mmol/L lower non-HDL cholesterol. Adjustment was for age and sex. ANGPTL4=angiopoietin like 4; CETP=cholesteryl ester transfer protein; CI = confidence interval; HMGCR= β-Hydroxy; LPL=lipoprotein lipase; NPC1L1=Nieman pick C1-like 1; HR = hazard ratio; PCSK9 = Proprotein convertase subtilisin/kexin type 9.

.

| **exposure** | **outcome** | **b** | **se** | **HR** | **CI low** | **CI high** |
| --- | --- | --- | --- | --- | --- | --- |
| ANGPTL4 | AD | -0.65 | 0.50 | 0.52 | 0.19 | 1.40 |
| ANGPTL4 | AllD | -0.69 | 0.35 | 0.50 | 0.25 | 1.00 |
| ANGPTL4 | IHD | -0.58 | 0.19 | 0.56 | 0.39 | 0.81 |
| ANGPTL4 | UD | -0.65 | 0.47 | 0.52 | 0.21 | 1.32 |
| ANGPTL4 | VD | -1.35 | 0.75 | 0.26 | 0.06 | 1.12 |
| CETP | AD | -2.44 | 1.26 | 0.09 | 0.01 | 1.03 |
| CETP | AllD | -1.29 | 0.88 | 0.27 | 0.05 | 1.55 |
| CETP | IHD | -0.31 | 0.47 | 0.74 | 0.29 | 1.85 |
| CETP | UD | -0.47 | 1.19 | 0.62 | 0.06 | 6.42 |
| CETP | VD | 0.12 | 1.76 | 1.13 | 0.04 | 35.76 |
| HMGCR | AD | -2.16 | 1.20 | 0.12 | 0.01 | 1.22 |
| HMGCR | AllD | -2.04 | 0.84 | 0.13 | 0.03 | 0.67 |
| HMGCR | IHD | -0.85 | 0.45 | 0.43 | 0.17 | 1.04 |
| HMGCR | UD | -2.49 | 1.12 | 0.08 | 0.01 | 0.75 |
| HMGCR | VD | -1.54 | 1.68 | 0.21 | 0.01 | 5.75 |
| LPL | AD | -0.04 | 0.26 | 0.96 | 0.58 | 1.60 |
| LPL | AllD | 0.02 | 0.18 | 1.02 | 0.72 | 1.45 |
| LPL | IHD | -0.33 | 0.10 | 0.72 | 0.59 | 0.87 |
| LPL | UD | 0.07 | 0.24 | 1.07 | 0.67 | 1.72 |
| LPL | VD | -0.02 | 0.36 | 0.98 | 0.49 | 1.97 |
| NPC1L1 | AD | 2.43 | 3.10 | 11.40 | 0.03 | 4935.36 |
| NPC1L1 | AllD | -0.13 | 2.13 | 0.88 | 0.01 | 57.10 |
| NPC1L1 | IHD | -0.10 | 1.13 | 0.91 | 0.10 | 8.37 |
| NPC1L1 | UD | -3.11 | 2.83 | 0.04 | 0.00 | 11.47 |
| NPC1L1 | VD | -0.19 | 4.21 | 0.83 | 0.00 | 3197.09 |
| PCSK9 | AD | -0.20 | 0.58 | 0.82 | 0.26 | 2.56 |
| PCSK9 | AllD | 0.00 | 0.40 | 1.00 | 0.46 | 2.21 |
| PCSK9 | IHD | -0.45 | 0.22 | 0.64 | 0.42 | 0.97 |
| PCSK9 | UD | -0.43 | 0.54 | 0.65 | 0.22 | 1.89 |
| PCSK9 | VD | 0.14 | 0.80 | 1.15 | 0.24 | 5.47 |

Supplementary Table 9. Cox regression: Risk of dementia and ischemic heart disease in UK Biobank. Betas and standard errors (se) are from Cox regression models. The allele scores were calculated based on the allele frequency of the variants in the UK Biobank. Change in the risk of dementia and ischemic heart disease is per 1 mmol/L lower non-HDL cholesterol. Adjustment was for age, sex, and first 10 principal components. ANGPTL4=angiopoietin like 4; CETP=cholesteryl ester transfer protein; HMGCR= β-Hydroxy β-methylglutaryl-CoA reductase; HR=hazard ratio; LPL=lipoprotein lipase; NPC1L1=Nieman pick C1-like 1; PCSK9 = Proprotein convertase subtilisin/kexin type 9.

.

|  | **Vascular dementia**  Sargan statistic (p-value) | | **Unspecified dementia**  Sargan statistic (p-value) | | **Alzheimer’s disease**  Sargan statistic (p-value) | | **All-cause dementia**  Sargan statistic (p-value) | | **Ischemic heart disease**  Sargan statistic (p-value) | |
| --- | --- | --- | --- | --- | --- | --- | --- | --- | --- | --- |
| **Gene** | **CCHS+CGPS** | **UKB** | **CCHS+CGPS** | **UKB** | **CCHS+CGPS** | **UKB** | **CCHS+CGPS** | **UKB** | **CCHS+CGPS** | **UKB** |
| **HMGCR** | NA | NA | NA | NA | NA | NA | NA | NA | NA | NA |
| **NPC1L1** | NA | NA | NA | NA | NA | NA | NA | NA | NA | NA |
| **PCSK9** | 0.31  (0.85) | 1.02  (0.60) | 0.33  (0.85) | 2.78  (0.25) | 0.13  (0.94) | 1.32  (0.52) | 0.07  (0.97) | 1.60  (0.45) | 4.1  (0.13) | 0.63  (0.73) |
| **ANGPTL4** | NA | NA | NA | NA | NA | NA | NA | NA | NA | NA |
| **LPL** | NA | NA | NA | NA | NA | NA | NA | NA | NA | NA |
| **CETP** | 3.468  (0.177) | 1.49  (0.48) | 51.7  (6*10^-12^) | 0.06  (0.97) | 58  (2*10^-13^) | 0.76  (0.69) | 29  (7*10^-7^) | 0.45  (0.80) | 10  (0.07) | 1.38  (0.50) |

Supplementary Table 10. Sargan Statistics for genetic instruments with more than one variant used in main analyses.

Sargan Statistics were calculated for drug targets with more than one genetic instrument using the ivreg package (<https://cran.r-project.org/web/packages/ivreg/vignettes/ivreg.html>). ANGPTL4=angiopoietin like 4; CETP=cholesteryl ester transfer protein; CCHS = Copenhagen City Heart Study; CGPS=Copenhagen General Population Study; HMGCR= β-hydroxy β-methylglutaryl-CoA reductase; HR=hazard ratio; LPL=lipoprotein lipase; NPC1L1=Nieman pick C1-like 1; PCSK9 = Proprotein convertase subtilisin/kexin type 9..

| **exposure** | **outcome** | **b** | **se** | **OR** | **CI low** | **Ci high** |
| --- | --- | --- | --- | --- | --- | --- |
| ANGPTL4 | AD | 1.03 | 0.76 | 2.80 | 0.63 | 12.42 |
| ANGPTL4 | AllD | 0.93 | 0.63 | 2.53 | 0.74 | 8.71 |
| ANGPTL4 | IHD | -1.01 | 0.42 | 0.36 | 0.16 | 0.83 |
| ANGPTL4 | UD | 0.18 | 0.85 | 1.20 | 0.23 | 6.33 |
| ANGPTL4 | VD | 1.40 | 1.72 | 4.05 | 0.14 | 117.02 |
| CETP | AD | -0.38 | 0.08 | 0.69 | 0.58 | 0.81 |
| CETP | AllD | -1.22 | 0.07 | 0.30 | 0.26 | 0.34 |
| CETP | IHD | -0.83 | 0.05 | 0.44 | 0.40 | 0.48 |
| CETP | UD | -1.95 | 0.09 | 0.14 | 0.12 | 0.17 |
| CETP | VD | -1.20 | 0.16 | 0.30 | 0.22 | 0.41 |
| HMGCR | AD | -0.74 | 0.19 | 0.48 | 0.33 | 0.69 |
| HMGCR | AllD | -1.69 | 0.16 | 0.18 | 0.13 | 0.25 |
| HMGCR | IHD | -1.02 | 0.11 | 0.36 | 0.29 | 0.44 |
| HMGCR | UD | -2.88 | 0.23 | 0.06 | 0.04 | 0.09 |
| HMGCR | VD | -1.10 | 0.43 | 0.33 | 0.14 | 0.77 |
| LPL | AD | 0.78 | 0.69 | 2.18 | 0.56 | 8.44 |
| LPL | AllD | 0.96 | 0.57 | 2.61 | 0.85 | 7.98 |
| LPL | IHD | -1.26 | 0.35 | 0.28 | 0.14 | 0.57 |
| LPL | UD | -0.78 | 0.62 | 0.46 | 0.14 | 1.53 |
| LPL | VD | -0.37 | 1.27 | 0.69 | 0.06 | 8.33 |
| NPC1L1 | AD | -0.45 | 0.22 | 0.64 | 0.42 | 0.98 |
| NPC1L1 | AllD | -1.80 | 0.19 | 0.17 | 0.11 | 0.24 |
| NPC1L1 | IHD | -0.93 | 0.12 | 0.39 | 0.31 | 0.50 |
| NPC1L1 | UD | -3.49 | 0.27 | 0.03 | 0.02 | 0.05 |
| NPC1L1 | VD | -1.49 | 0.48 | 0.22 | 0.09 | 0.57 |
| PCSK9 | AD | 0.137 | 0.308 | 1.146828 | 0.627081 | 2.097361 |
| PCSK9 | AllD | -0.0516 | 0.26 | 0.949709 | 0.570524 | 1.580909 |
| PCSK9 | IHD | -0.44 | 0.16 | 0.644036 | 0.470669 | 0.881262 |
| PCSK9 | UD | -2.52 | 3.70E-01 | 0.08046 | 0.038961 | 0.166161 |
| PCSK9 | VD | 0.057 | 7.36E-01 | 1.058656 | 0.250184 | 4.479718 |

Supplementary Table 11. One-sample MR using restricted variants: Risk of dementia and ischemic heart disease in CCHS+CGPS. Betas and standard errors (se) are from on-sample Mendelian randomization analyses. Genetic variants were used as individual instruments. Change in the risk of dementia and ischemic heart disease is per 1 mmol/L lower non-HDL cholesterol. Adjustment was for age and sex. All variants available in CCHS+CGPS included in analyses. ANGPTL4=angiopoietin like 4; CETP=cholesteryl ester transfer protein; HMGCR= β-Hydroxy β-methylglutaryl-CoA reductase; HR=hazard ratio; LPL=lipoprotein lipase; NPC1L1=Nieman pick C1-like 1; PCSK9 = Proprotein convertase subtilisin/kexin type 9.

| **exposure** | **outcome** | **b** | **se** | **OR** | **CI low** | **CI high** |
| --- | --- | --- | --- | --- | --- | --- |
| ANGPTL4 | AD | -3.17 | 2.56 | 0.04 | 0.00 | 6.35 |
| ANGPTL4 | AllD | -3.45 | 1.87 | 0.03 | 0.00 | 1.23 |
| ANGPTL4 | IHD | -3.26 | 1.09 | 0.04 | 0.00 | 0.33 |
| ANGPTL4 | UD | -3.32 | 2.39 | 0.04 | 0.00 | 3.93 |
| ANGPTL4 | VD | -6.49 | 3.98 | 0.00 | 0.00 | 3.72 |
| CETP | AD | -1.31 | 0.79 | 0.27 | 0.06 | 1.26 |
| CETP | AllD | -0.80 | 0.54 | 0.45 | 0.16 | 1.30 |
| CETP | IHD | -0.68 | 0.27 | 0.50 | 0.30 | 0.86 |
| CETP | UD | -0.36 | 0.71 | 0.70 | 0.17 | 2.81 |
| CETP | VD | -0.74 | 1.04 | 0.48 | 0.06 | 3.66 |
| HMGCR | AD | -0.58 | 0.50 | 0.56 | 0.21 | 1.48 |
| HMGCR | AllD | -0.32 | 0.35 | 0.73 | 0.37 | 1.43 |
| HMGCR | IHD | 0.10 | 0.17 | 1.11 | 0.79 | 1.54 |
| HMGCR | UD | -1.84 | 0.96 | 0.16 | 0.02 | 1.04 |
| HMGCR | VD | -1.08 | 1.44 | 0.34 | 0.02 | 5.68 |
| LPL | AD | -0.36 | 0.86 | 0.70 | 0.13 | 3.78 |
| LPL | AllD | -0.31 | 0.59 | 0.73 | 0.23 | 2.33 |
| LPL | IHD | -1.33 | 0.31 | 0.26 | 0.14 | 0.49 |
| LPL | UD | 0.28 | 0.84 | 1.33 | 0.25 | 6.93 |
| LPL | VD | 0.12 | 1.27 | 1.13 | 0.09 | 13.50 |
| NPC1L1 | AD | 0.89 | 1.05 | 2.43 | 0.31 | 18.92 |
| NPC1L1 | AllD | -0.74 | 0.72 | 0.48 | 0.12 | 1.95 |
| NPC1L1 | IHD | -0.69 | 0.36 | 0.50 | 0.25 | 1.01 |
| NPC1L1 | UD | -3.19 | 2.22 | 0.04 | 0.00 | 3.22 |
| NPC1L1 | VD | -0.46 | 3.23 | 0.63 | 0.00 | 359.13 |
| PCSK9 | AD | 0.35 | 0.30 | 1.41 | 0.79 | 2.54 |
| PCSK9 | AllD | -0.01 | 0.22 | 0.99 | 0.65 | 1.52 |
| PCSK9 | IHD | -0.52 | 0.12 | 0.60 | 0.48 | 0.75 |
| PCSK9 | UD | -0.30 | 0.30 | 0.74 | 0.41 | 1.34 |
| PCSK9 | VD | -0.46 | 0.46 | 0.63 | 0.25 | 1.56 |

Supplementary Table 12. One-sample MR using restricted number of variants: Risk of dementia and ischemic heart disease in UK Biobank. Betas and standard errors (se) are from one-sampel MR analyses. Genetic variants were used as individual instruments. Change in the risk of dementia and ischemic heart disease is per 1 mmol/L lower non-HDL cholesterol. Adjustment was for age and sex. Variants with of R2 ≤0.05 included in analyses. ANGPTL4=angiopoietin like 4; CETP=cholesteryl ester transfer protein; HMGCR= β-Hydroxy β-methylglutaryl-CoA reductase; HR=hazard ratio; LPL=lipoprotein lipase; NPC1L1=Nieman pick C1-like 1; PCSK9 = Proprotein convertase subtilisin/kexin type 9.

| outcome | exposure | method | nsnp | b | se | OR | CI low | CI high |
| --- | --- | --- | --- | --- | --- | --- | --- | --- |
| AD | CETP | Wald ratio | 1 | 0.175263 | 0.584914 | 1.19156 | 0.37864 | 3.749769 |
| AllD | CETP | Wald ratio | 1 | -0.41599 | 0.475111 | 0.65969 | 0.259967 | 1.674025 |
| IHD | CETP | Wald ratio | 1 | -0.88159 | 0.291401 | 0.414122 | 0.233928 | 0.733119 |
| UD | CETP | Wald ratio | 1 | -0.13297 | 0.648262 | 0.875493 | 0.24572 | 3.119354 |
| VD | CETP | Wald ratio | 1 | -2.25941 | 1.321863 | 0.104412 | 0.007826 | 1.392969 |
| AD | LPL | Wald ratio | 1 | 0.256863 | 0.660289 | 1.292868 | 0.354409 | 4.716327 |
| AllD | LPL | Wald ratio | 1 | 0.306725 | 0.539413 | 1.358967 | 0.472119 | 3.911706 |
| IHD | LPL | Wald ratio | 1 | -0.48351 | 0.3309 | 0.616617 | 0.322365 | 1.179461 |
| UD | LPL | Wald ratio | 1 | -0.79628 | 0.764546 | 0.451005 | 0.100783 | 2.018256 |
| VD | LPL | Wald ratio | 1 | -0.46991 | 1.526069 | 0.625059 | 0.031398 | 12.44336 |
| AD | NPC1L1 | Wald ratio | 1 | -2.25742 | 1.610012 | 0.10462 | 0.004458 | 2.455176 |
| AllD | NPC1L1 | Wald ratio | 1 | -1.88935 | 1.297659 | 0.15117 | 0.011882 | 1.923335 |
| IHD | NPC1L1 | Wald ratio | 1 | -0.13154 | 0.822782 | 0.876741 | 0.174785 | 4.39782 |
| UD | NPC1L1 | Wald ratio | 1 | -3.13368 | 1.816723 | 0.043557 | 0.001238 | 1.532798 |
| VD | NPC1L1 | Wald ratio | 1 | -0.9142 | 3.778702 | 0.400836 | 0.000243 | 659.8774 |
| AD | HMGCR | Wald ratio | 1 | -0.76338 | 0.766821 | 0.46609 | 0.10369 | 2.095082 |
| AllD | HMGCR | Wald ratio | 1 | -0.86743 | 0.638474 | 0.420029 | 0.120171 | 1.46811 |
| IHD | HMGCR | Wald ratio | 1 | -1.10872 | 0.380691 | 0.329982 | 0.156473 | 0.69589 |
| UD | HMGCR | Wald ratio | 1 | -0.54022 | 0.906408 | 0.582621 | 0.098591 | 3.442991 |
| VD | HMGCR | Wald ratio | 1 | -0.17929 | 1.812816 | 0.835866 | 0.023935 | 29.19016 |
| AD | ANGPTl4 | Wald ratio | 1 | 1.345241 | 1.06504 | 3.839113 | 0.476048 | 30.96071 |
| AllD | ANGPTl4 | Wald ratio | 1 | 1.082722 | 0.873523 | 2.952706 | 0.532921 | 16.3598 |
| IHD | ANGPTL4 | Wald ratio | 1 | -1.63224 | 0.572646 | 0.19549 | 0.063633 | 0.60058 |
| UD | ANGPTL4 | Wald ratio | 1 | -0.24892 | 1.235065 | 0.779644 | 0.069277 | 8.774151 |
| VD | ANGPTL4 | Wald ratio | 1 | 1.441816 | 2.374916 | 4.228367 | 0.040235 | 444.3669 |
| AD | PCSK9 | Weighted median | 3 | -0.12119 | 0.215197 | 0.88587 | 0.58102 | 1.350669 |
| AllD | PCSK9 | Weighted median | 3 | -0.30233 | 0.140383 | 0.739096 | 0.561311 | 0.97319 |
| IHD | PCSK9 | Weighted median | 3 | -0.19128 | 0.620285 | 0.825904 | 0.244868 | 2.78565 |
| UD | PCSK9 | Weighted median | 3 | 0.100142 | 0.251936 | 1.105328 | 0.674588 | 1.811103 |
| VD | PCSK9 | Weighted median | 3 | -0.34013 | 0.327438 | 0.711679 | 0.374596 | 1.352088 |

Supplementary Table 13. Two-sample MR using restricted number of variants: Risk of dementia and ischemic heart disease in CCHS+CGPS. Betas and standard errors (se) are from two-sample Mendelian randomization analyses. Genetic variants were used as individual instruments. Change in the risk of dementia and ischemic heart disease is per 1 mmol/L lower non-HDL cholesterol. Adjustment was for age and sex. Variants with of R2 ≤0.05 included in analyses. ANGPTL4=angiopoietin like 4; CETP=cholesteryl ester transfer protein; HMGCR= β-Hydroxy β-methylglutaryl-CoA reductase; HR=hazard ratio; LPL=lipoprotein lipase; NPC1L1=Nieman pick C1-like 1; PCSK9 = Proprotein convertase subtilisin/kexin type 9.

| **outcome** | **exposure** | **method** | **nsnp** | **b** | **se** | **OR** | **CI low** | **CI high** |
| --- | --- | --- | --- | --- | --- | --- | --- | --- |
| AD | ANGPTL4 | Wald ratio | 1 | -1.95645 | 1.552837 | 0.14136 | 0.006738 | 2.9657 |
| AllD | ANGPTL4 | Wald ratio | 1 | -2.13206 | 1.082548 | 0.118593 | 0.014209 | 0.989791 |
| IHD | ANGPTL4 | Wald ratio | 1 | -2.01594 | 0.524195 | 0.133195 | 0.047674 | 0.372129 |
| UD | ANGPTL4 | Wald ratio | 1 | -2.05169 | 1.432898 | 0.128518 | 0.007749 | 2.131433 |
| VD | ANGPTL4 | Wald ratio | 1 | -4.01105 | 2.291221 | 0.018114 | 0.000203 | 1.615652 |
| AD | CETP | Weighted median | 3 | -0.99108 | 0.569358 | 0.371175 | 0.121599 | 1.132989 |
| AllD | CETP | Weighted median | 3 | -0.60005 | 0.388989 | 0.548786 | 0.256029 | 1.176299 |
| IHD | CETP | Weighted median | 3 | -0.50895 | 0.195311 | 0.601127 | 0.409934 | 0.881493 |
| UD | CETP | Weighted median | 3 | -0.2752 | 0.521639 | 0.759421 | 0.273184 | 2.111109 |
| VD | CETP | Weighted median | 3 | -0.24614 | 1.518516 | 0.781815 | 0.039858 | 15.33527 |
| AD | HMGCR | Wald ratio | 1 | -1.49054 | 0.892558 | 0.22525 | 0.039166 | 1.295465 |
| AllD | HMGCR | Wald ratio | 1 | -1.35532 | 0.621741 | 0.257866 | 0.076235 | 0.87223 |
| IHD | HMGCR | Wald ratio | 1 | -0.49553 | 0.310229 | 0.609249 | 0.331683 | 1.119096 |
| UD | HMGCR | Wald ratio | 1 | -1.5891 | 0.817741 | 0.204109 | 0.041095 | 1.013767 |
| VD | HMGCR | Wald ratio | 1 | -0.93223 | 1.241715 | 0.393674 | 0.034528 | 4.488555 |
| AD | LPL | Wald ratio | 1 | -0.04513 | 0.743864 | 0.955877 | 0.22244 | 4.107631 |
| AllD | LPL | Wald ratio | 1 | 0.112625 | 0.514313 | 1.119213 | 0.408433 | 3.066933 |
| IHD | LPL | Wald ratio | 1 | -0.95053 | 0.257295 | 0.386536 | 0.23344 | 0.640036 |
| UD | LPL | Wald ratio | 1 | 0.225186 | 0.678675 | 1.252556 | 0.331205 | 4.736926 |
| VD | LPL | Wald ratio | 1 | 0.09743 | 1.019611 | 1.102334 | 0.149418 | 8.132487 |
| AD | NPC1L1 | Wald ratio | 1 | 1.284657 | 1.881839 | 3.613429 | 0.090379 | 144.4686 |
| AllD | NPC1L1 | Wald ratio | 1 | -0.57134 | 1.29223 | 0.564768 | 0.044864 | 7.109484 |
| IHD | NPC1L1 | Wald ratio | 1 | -0.10313 | 0.634563 | 0.902008 | 0.260052 | 3.12868 |
| UD | NPC1L1 | Wald ratio | 1 | -2.53839 | 1.691315 | 0.078993 | 0.00287 | 2.17403 |
| VD | NPC1L1 | Wald ratio | 1 | -0.36211 | 2.559923 | 0.696206 | 0.00461 | 105.1449 |
| AD | PCSK9 | Weighted median | 3 | 0.245818 | 0.239335 | 1.278667 | 0.799892 | 2.044011 |
| AllD | PCSK9 | Weighted median | 3 | 0.035723 | 0.17887 | 1.036368 | 0.729889 | 1.471538 |
| IHD | PCSK9 | Weighted median | 3 | -0.42027 | 0.093601 | 0.656872 | 0.546772 | 0.789143 |
| UD | PCSK9 | Weighted median | 3 | 0.245818 | 0.245552 | 1.278667 | 0.790204 | 2.06907 |
| VD | PCSK9 | Weighted median | 3 | 0.245818 | 0.236208 | 1.278667 | 0.80481 | 2.031521 |

Supplementary Table 14. Two-sample MR using restricted number of variants: Risk of dementia and ischemic heart disease in UK Biobank. Betas and standard errors (se) are from two-sample Mendelian randomization analyses. Genetic variants were used as individual instruments. Change in the risk of dementia and ischemic heart disease is per 1 mmol/L lower non-HDL cholesterol. Adjustment was for age and sex. Variants with of R2 ≤0.05 included in analyses. ANGPTL4=angiopoietin like 4; CETP=cholesteryl ester transfer protein; HMGCR= β-Hydroxy β-methylglutaryl-CoA reductase; HR=hazard ratio; LPL=lipoprotein lipase; NPC1L1=Nieman pick C1-like 1; PCSK9 = Proprotein convertase subtilisin/kexin type 9.

| **outcome** | **exposure** | **method** | **nsnp** | **b** | **se** | **OR** | **CI low** | **CI high** |
| --- | --- | --- | --- | --- | --- | --- | --- | --- |
| AD | ANGPTL4 | Wald ratio | 1 | 0.932537 | 0.81558 | 2.540947 | 0.51376 | 12.56698 |
| AllD | ANGPTL4 | Wald ratio | 1 | 0.202469 | 0.480863 | 1.224422 | 0.477103 | 3.142319 |
| IHD | ANGPTL4 | Wald ratio | 1 | -1.2781 | 0.286989 | 0.278566 | 0.158722 | 0.488899 |
| UD | ANGPTL4 | Wald ratio | 1 | -1.80962 | 0.944063 | 0.163717 | 0.025733 | 1.04159 |
| VD | ANGPTL4 | Wald ratio | 1 | 0.386282 | 1.157622 | 1.471499 | 0.152185 | 14.22812 |
| AD | CETP | Weighted median | 3 | 0.392945 | 0.392653 | 1.481337 | 0.686152 | 3.198065 |
| AllD | CETP | Weighted median | 3 | -0.10247 | 0.252997 | 0.902608 | 0.549723 | 1.482021 |
| IHD | CETP | Weighted median | 3 | -0.51618 | 0.134741 | 0.596794 | 0.45828 | 0.777174 |
| UD | CETP | Weighted median | 3 | 0.010866 | 0.435516 | 1.010925 | 0.430527 | 2.373762 |
| VD | CETP | Weighted median | 3 | -0.39279 | 0.578159 | 0.675169 | 0.217407 | 2.096775 |
| AD | HMGCR | Wald ratio | 1 | 0.433676 | 0.565095 | 1.542919 | 0.509712 | 4.670479 |
| AllD | HMGCR | Wald ratio | 1 | -0.19662 | 0.337204 | 0.821503 | 0.424205 | 1.590899 |
| IHD | HMGCR | Wald ratio | 1 | -0.99822 | 0.195964 | 0.368534 | 0.250998 | 0.54111 |
| UD | HMGCR | Wald ratio | 1 | -0.42152 | 0.650415 | 0.65605 | 0.183355 | 2.347369 |
| VD | HMGCR | Wald ratio | 1 | -0.10249 | 0.821566 | 0.902588 | 0.180367 | 4.516696 |
| AD | LPL | Wald ratio | 1 | 0.705674 | 0.52031 | 2.025211 | 0.730422 | 5.615221 |
| AllD | LPL | Wald ratio | 1 | 0.652919 | 0.30711 | 1.92114 | 1.052305 | 3.507329 |
| IHD | LPL | Wald ratio | 1 | -0.70866 | 0.18109 | 0.492304 | 0.345213 | 0.70207 |
| UD | LPL | Wald ratio | 1 | -0.15437 | 0.594226 | 0.856956 | 0.267389 | 2.746462 |
| VD | LPL | Wald ratio | 1 | 0.806602 | 0.747333 | 2.240282 | 0.517798 | 9.692708 |
| AD | NPC1L1 | Wald ratio | 1 | -0.66188 | 1.260888 | 0.515879 | 0.043577 | 6.107136 |
| AllD | NPC1L1 | Wald ratio | 1 | -0.59405 | 0.746136 | 0.552085 | 0.127903 | 2.383031 |
| IHD | NPC1L1 | Wald ratio | 1 | -0.68195 | 0.437231 | 0.505628 | 0.214611 | 1.191268 |
| UD | NPC1L1 | Wald ratio | 1 | -1.36373 | 1.432595 | 0.255705 | 0.015427 | 4.238275 |
| VD | NPC1L1 | Wald ratio | 1 | 1.167543 | 1.81847 | 3.214085 | 0.091022 | 113.4933 |
| AD | PCSK9 | Weighted median | 3 | 0.107426 | 0.119793 | 1.113408 | 0.88041 | 1.408069 |
| AllD | PCSK9 | Weighted median | 3 | 0.04659 | 0.077333 | 1.047693 | 0.90034 | 1.219162 |
| IHD | PCSK9 | Weighted median | 3 | -0.49966 | 0.046873 | 0.606739 | 0.553481 | 0.665121 |
| UD | PCSK9 | Weighted median | 3 | 0.086445 | 0.143061 | 1.090291 | 0.823695 | 1.443174 |
| VD | PCSK9 | Weighted median | 3 | 0.096233 | 0.186013 | 1.101016 | 0.764638 | 1.585372 |

Supplementary Table 15. Two-sample MR using restricted number of variants: Risk of dementia and ischemic heart disease in FinnGen. Betas and standard errors (se) are from two-sample Mendelian randomization analyses. Genetic variants were used as individual instruments. Change in the risk of dementia and ischemic heart disease is per 1 mmol/L lower non-HDL cholesterol. Adjustment was for age and sex. Variants with of R2 ≤0.05 included in analyses. ANGPTL4=angiopoietin like 4; CETP=cholesteryl ester transfer protein; HMGCR= β-Hydroxy β-methylglutaryl-CoA reductase; HR=hazard ratio; LPL=lipoprotein lipase; NPC1L1=Nieman pick C1-like 1; PCSK9 = Proprotein convertase subtilisin/kexin type 9.

| outcome | exposure | egger_intercept | se | pval | cohort |
| --- | --- | --- | --- | --- | --- |
| Vascular dementia | CETP | 0.497444 | 0.353297 | 0.393148 | CCHS+CHPS |
| Unspecified dementia | CETP | 0.766006 | 0.750356 | 0.49343 | CCHS+CHPS |
| Alzheimer's disease | CETP | 0.149577 | 0.168308 | 0.537469 | CCHS+CHPS |
| All-cause dementia | CETP | 0.473944 | 0.470594 | 0.497742 | CCHS+CHPS |
| Ischemic heart disease | CETP | 0.319512 | 0.297971 | 0.477801 | CCHS+CHPS |
| Vascular dementia | CETP | -0.08045 | 0.163415 | 0.708773 | UKB |
| Unspecified dementia | CETP | -0.0139 | 0.08298 | 0.894338 | UKB |
| Alzheimer's disease | CETP | 0.07614 | 0.0879 | 0.545562 | UKB |
| All-cause dementia | CETP | 0.044161 | 0.061696 | 0.604503 | UKB |
| Ischemic heart disease | CETP | -0.023 | 0.030725 | 0.590884 | UKB |
| Vascular dementia | CETP | -0.09679 | 0.124959 | 0.580449 | FinnGen |
| Unspecified dementia | CETP | -0.10671 | 0.111919 | 0.515166 | FinnGen |
| Alzheimer's disease | CETP | 0.117187 | 0.111138 | 0.483138 | FinnGen |
| All-cause dementia | CETP | -0.02807 | 0.105885 | 0.83502 | FinnGen |
| Ischemic heart disease | CETP | -0.02462 | 0.063115 | 0.763212 | FinnGen |
| Vascular dementia | PCSK9 | 0.01691739 | 0.09814693 | 0.8913349 | CCHS+CHPS |
| Unspecified dementia | PCSK9 | -0.02546582 | 0.045725 | 0.6765008 | CCHS+CHPS |
| Alzheimer's disease | PCSK9 | 0.006569281 | 0.04173368 | 0.9006055 | CCHS+CHPS |
| All-cause dementia | PCSK9 | -0.004357148 | 0.0346936 | 0.9204637 | CCHS+CHPS |
| Ischemic heart disease | PCSK9 | -0.01007935 | 0.03573707 | 0.8249925 | CCHS+CHPS |
| Vascular dementia | PCSK9 | -0.05678507 | 0.05919616 | 0.5132326 | UKB |
| Unspecified dementia | PCSK9 | 0.01205283 | 0.06288514 | 0.8794448 | UKB |
| Alzheimer's disease | PCSK9 | 0.05283625 | 0.042828 | 0.4336392 | UKB |
| All-cause dementia | PCSK9 | 0.002787276 | 0.03181905 | 0.9443756 | UKB |
| Ischemic heart disease | PCSK9 | -0.009293346 | 0.01474601 | 0.641997 | UKB |
| Vascular dementia | PCSK9 | -0.02198005 | 0.04519088 | 0.7118058 | FinnGen |
| Unspecified dementia | PCSK9 | -0.004571097 | 0.04067288 | 0.9287513 | FinnGen |
| Alzheimer's disease | PCSK9 | -0.03778207 | 0.02689523 | 0.3938359 | FinnGen |
| All-cause dementia | PCSK9 | -0.01947245 | 0.01602879 | 0.4384397 | FinnGen |
| Ischemic heart disease | PCSK9 | 0.02328933 | 0.0282927 | 0.5615585 | FinnGen |

Supplementary Table 16. Analyses of pleiotropy in two-sample MR analyses.

Analyses were performed using the “TwoSampleMR” R package. CETP=cholesteryl ester transfer protein; CCHS = Copenhagen City Heart Study; CGPS=Copenhagen General Population Study; PCSK9 = Proprotein convertase subtilisin/kexin type 9.

| outcome | exposure | method | Q | Q_df | Q_pval | cohort |
| --- | --- | --- | --- | --- | --- | --- |
| Vascular dementia | CETP | MR Egger | 15.40663 | 1 | 8.67E-05 | CCHS+CPGS |
| Vascular dementia | CETP | Inverse variance weighted | 45.94991 | 2 | 1.05E-10 | CCHS+CPGS |
| Unspecified dementia | CETP | MR Egger | 316.5053 | 1 | 8.36E-71 | CCHS+CPGS |
| Unspecified dementia | CETP | Inverse variance weighted | 646.3514 | 2 | 4.43E-141 | CCHS+CPGS |
| Alzheimer's disease | CETP | MR Egger | 13.67396 | 1 | 0.000217 | CCHS+CPGS |
| Alzheimer's disease | CETP | Inverse variance weighted | 24.47371 | 2 | 4.85E-06 | CCHS+CPGS |
| All-cause dementia | CETP | MR Egger | 190.5048 | 1 | 2.47E-43 | CCHS+CPGS |
| All-cause dementia | CETP | Inverse variance weighted | 383.7313 | 2 | 4.72E-84 | CCHS+CPGS |
| Ischemic heart disease | CETP | MR Egger | 149.5108 | 1 | 2.22E-34 | CCHS+CPGS |
| Ischemic heart disease | CETP | Inverse variance weighted | 321.4196 | 2 | 1.60E-70 | CCHS+CPGS |
| Vascular dementia | CETP | MR Egger | 1.597938 | 1 | 0.206196 | UKB |
| Vascular dementia | CETP | Inverse variance weighted | 1.985195 | 2 | 0.370613 | UKB |
| Unspecified dementia | CETP | MR Egger | 0.00549 | 1 | 0.940937 | UKB |
| Unspecified dementia | CETP | Inverse variance weighted | 0.033551 | 2 | 0.983365 | UKB |
| Alzheimer's disease | CETP | MR Egger | 0.121021 | 1 | 0.72793 | UKB |
| Alzheimer's disease | CETP | Inverse variance weighted | 0.871339 | 2 | 0.646831 | UKB |
| All-cause dementia | CETP | MR Egger | 0.029417 | 1 | 0.863821 | UKB |
| All-cause dementia | CETP | Inverse variance weighted | 0.541771 | 2 | 0.762704 | UKB |
| Ischemic heart disease | CETP | MR Egger | 0.369708 | 1 | 0.543163 | UKB |
| Ischemic heart disease | CETP | Inverse variance weighted | 0.930188 | 2 | 0.628076 | UKB |
| Vascular dementia | CETP | MR Egger | 0.037962 | 1 | 0.845519 | FinnGen |
| Vascular dementia | CETP | Inverse variance weighted | 0.63789 | 2 | 0.726916 | FinnGen |
| Unspecified dementia | CETP | MR Egger | 1.279424 | 1 | 0.258006 | FinnGen |
| Unspecified dementia | CETP | Inverse variance weighted | 2.442518 | 2 | 0.294859 | FinnGen |
| Alzheimer's disease | CETP | MR Egger | 1.662664 | 1 | 0.197244 | FinnGen |
| Alzheimer's disease | CETP | Inverse variance weighted | 3.511238 | 2 | 0.1728 | FinnGen |
| All-cause dementia | CETP | MR Egger | 4.23601 | 1 | 0.039575 | FinnGen |
| All-cause dementia | CETP | Inverse variance weighted | 4.533735 | 2 | 0.103636 | FinnGen |
| Ischemic heart disease | CETP | MR Egger | 4.341523 | 1 | 0.037194 | FinnGen |
| Ischemic heart disease | CETP | Inverse variance weighted | 5.002216 | 2 | 0.081994 | FinnGen |

Supplementary Table 17. Analyses of heterogeneity in two-sample MR analyses. Analyses were performed using the “TwoSampleMR” R package. CETP=cholesteryl ester transfer protein; CCHS = Copenhagen City Heart Study; CGPS=Copenhagen General Population Study.

| outcome | exposure | method | Q | Q_df | Q_pval | cohort |
| --- | --- | --- | --- | --- | --- | --- |
| Vascular dementia | PCSK9 | MR Egger | 0.01350079 | 1 | 0.9074997 | CCHS+CPGS |
| Vascular dementia | PCSK9 | Inverse variance weighted | 0.04321152 | 2 | 0.9786260 | CCHS+CPGS |
| Unspecified dementia | PCSK9 | MR Egger | 0.05376428 | 1 | 0.8166380 | CCHS+CPGS |
| Unspecified dementia | PCSK9 | Inverse variance weighted | 0.36394006 | 2 | 0.8336263 | CCHS+CPGS |
| Alzheimer's disease | PCSK9 | MR Egger | 0.02383588 | 1 | 0.8773032 | CCHS+CPGS |
| Alzheimer's disease | PCSK9 | Inverse variance weighted | 0.04861366 | 2 | 0.9759862 | CCHS+CPGS |
| All-cause dementia | PCSK9 | MR Egger | 0.05308538 | 1 | 0.8177789 | CCHS+CPGS |
| All-cause dementia | PCSK9 | Inverse variance weighted | 0.06885808 | 2 | 0.9661569 | CCHS+CPGS |
| Ischemic heart disease | PCSK9 | MR Egger | 2.897454 | 1 | 0.0887196 | CCHS+CPGS |
| Ischemic heart disease | PCSK9 | Inverse variance weighted | 3.127939 | 2 | 0.2093036 | CCHS+CPGS |
| Vascular dementia | PCSK9 | MR Egger | 0.04593274 | 1 | 0.8302981 | UKB |
| Vascular dementia | PCSK9 | Inverse variance weighted | 0.96613050 | 2 | 0.6168896 | UKB |
| Unspecified dementia | PCSK9 | MR Egger | 2.553470 | 1 | 0.1100523 | UKB |
| Unspecified dementia | PCSK9 | Inverse variance weighted | 2.647273 | 2 | 0.2661657 | UKB |
| Alzheimer's disease | PCSK9 | MR Egger | 0.08509935 | 1 | 0.7705023 | UKB |
| Alzheimer's disease | PCSK9 | Inverse variance weighted | 1.60707717 | 2 | 0.4477418 | UKB |
| All-cause dementia | PCSK9 | MR Egger | 1.150895 | 1 | 0.2833620 | UKB |
| All-cause dementia | PCSK9 | Inverse variance weighted | 1.159726 | 2 | 0.5599751 | UKB |
| Ischemic heart disease | PCSK9 | MR Egger | 0.6010571 | 1 | 0.4381750 | UKB |
| Ischemic heart disease | PCSK9 | Inverse variance weighted | 0.9982440 | 2 | 0.6070634 | UKB |
| Vascular dementia | PCSK9 | MR Egger | 1.347817 | 1 | 0.2456602 | FinnGen |
| Vascular dementia | PCSK9 | Inverse variance weighted | 1.666667 | 2 | 0.4345981 | FinnGen |
| Unspecified dementia | PCSK9 | MR Egger | 1.724124 | 1 | 0.1891630 | FinnGen |
| Unspecified dementia | PCSK9 | Inverse variance weighted | 1.745901 | 2 | 0.4177172 | FinnGen |
| Alzheimer's disease | PCSK9 | MR Egger | 0.9946245 | 1 | 0.3186147 | FinnGen |
| Alzheimer's disease | PCSK9 | Inverse variance weighted | 2.9680507 | 2 | 0.2267232 | FinnGen |
| All-cause dementia | PCSK9 | MR Egger | 0.3102562 | 1 | 0.5775230 | FinnGen |
| All-cause dementia | PCSK9 | Inverse variance weighted | 1.7860981 | 2 | 0.4094056 | FinnGen |
| Ischemic heart disease | PCSK9 | MR Egger | 8.989328 | 1 | 0.0027156091 | FinnGen |
| Ischemic heart disease | PCSK9 | Inverse variance weighted | 15.080379 | 2 | 0.0005312969 | FinnGen |

Supplementary Table 18. Analyses of heterogeneity in two-sample MR analyses. Analyses were performed using the “TwoSampleMR” R package. CCHS = Copenhagen City Heart Study; CGPS=Copenhagen General Population Study; PCSK9 = Proprotein convertase subtilisin/kexin type 9.

| **exposure** | **outcome** | **b** | **se** | **OR** | **CI low** | **CI high** |
| --- | --- | --- | --- | --- | --- | --- |
| ANGPTL4 | AD | 1.03 | 0.76 | 2.801066 | 0.631536 | 12.4236262 |
| ANGPTL4 | AllD | 0.93 | 0.63 | 2.534509 | 0.737271 | 8.71285917 |
| ANGPTL4 | IHD | -1.01 | 0.42 | 0.364219 | 0.159901 | 0.82960964 |
| ANGPTL4 | VaD | 1.3999 | 0.176 | 4.054794 | 2.871803 | 5.72509991 |
| ANGPTL4 | UD | 1.80E-01 | 8.50E-01 | 1.197217 | 0.226276 | 6.33443106 |
| CETP | AD | -3.96E-01 | 8.10E-02 | 0.673007 | 0.57421 | 0.78880195 |
| CETP | AllD | -1.18E+00 | 6.50E-02 | 0.307279 | 0.270523 | 0.34902909 |
| CETP | IHD | -8.10E-01 | 4.57E-01 | 0.444858 | 0.181642 | 1.08950123 |
| CETP | VaD | -1.14E+00 | 1.57E-01 | 0.319819 | 0.235106 | 0.43505623 |
| CETP | UD | -1.85E+00 | 8.00E-02 | 0.157237 | 0.134418 | 0.18393001 |
| HMGCR | AD | -0.477 | 0.14596 | 0.620643 | 0.466227 | 0.8262 |
| HMGCR | AllD | -1.15 | 0.12 | 0.316637 | 0.250274 | 0.40059674 |
| HMGCR | IHD | -0.857 | 0.0857 | 0.424433 | 0.358807 | 0.50206384 |
| HMGCR | VaD | -0.53 | 0.3 | 0.588605 | 0.326933 | 1.059715 |
| HMGCR | UD | -1.90E+00 | 5.30E-01 | 0.149718 | 0.052982 | 0.42307746 |
| LPL | AD | 0.35 | 0.399 | 1.419068 | 0.649183 | 3.10197809 |
| LPL | AllD | 0.63 | 0.32 | 1.877611 | 1.002804 | 3.51556411 |
| LPL | IHD | -0.563 | 0.2 | 0.569498 | 0.384812 | 0.84282157 |
| LPL | VaD | 6.10E-01 | 8.80E-01 | 1.840431 | 0.327981 | 10.3273942 |
| LPL | UD | 4.60E-01 | 4.20E-01 | 1.584074 | 0.695447 | 3.60816741 |
| NPC1L1 | AD | -0.44 | 0.215 | 0.644036 | 0.42257 | 0.98157191 |
| NPC1L1 | AllD | -1.86 | 0.19 | 0.155673 | 0.107271 | 0.2259142 |
| NPC1L1 | IHD | -0.975 | 0.116 | 0.377192 | 0.300484 | 0.47348265 |
| NPC1L1 | VaD | -0.41 | 0.4768 | 0.66365 | 0.260663 | 1.68966114 |
| NPC1L1 | UD | -3.55E+00 | 2.70E-01 | 0.028725 | 0.016921 | 0.04876219 |
| PCSK9 | AD | 0.106 | 0.35 | 1.111822 | 0.559898 | 2.207808 |
| PCSK9 | AllD | -0.59 | 8.60E-01 | 0.554327 | 0.102735 | 2.990977 |
| PCSK9 | IHD | -0.59 | 0.2 | 0.554327 | 0.374561 | 0.82037 |
| PCSK9 | UD | -0.196 | 0.295 | 0.822012 | 0.461072 | 1.465505 |
| PCSK9 | VD | -0.59 | 8.60E-01 | 0.554327 | 0.102735 | 2.990977 |

Supplementary Table 19. One-sample MR using all available variants: Risk of dementia and ischemic heart disease in CCHS+CGPS. Betas and standard errors (se) are from Cox regression models. The allele scores were calculated based on the allele frequency of the variants in the UK Biobank. Change in the risk of dementia and ischemic heart disease is per 1 mmol/L lower non-HDL cholesterol. Adjustment was for age and sex. All variants available in CCHS+CGPS included in analyses. ANGPTL4=angiopoietin like 4; CETP=cholesteryl ester transfer protein; CPH = Copenhagen studies; CI=confidence interval; HMGCR= β-Hydroxy β-methylglutaryl-CoA reductase; HR=hazard ratio; LPL=lipoprotein lipase; NPC1L1=Nieman pick C1-like 1; PCSK9 = Proprotein convertase subtilisin/kexin type 9. .

| **exposure** | **outcome** | **b** | **se** | **OR** | **CI lo** | **CI up** |
| --- | --- | --- | --- | --- | --- | --- |
| ANGPTL4 | AD | -3.17E+00 | 2.56E+00 | 0.042096 | 0.00027902 | 6.3510744 |
| ANGPTL4 | AllD | -3.45E+00 | 1.87E+00 | 0.031676 | 0.0008131 | 1.2339939 |
| ANGPTL4 | IHD | -3.26E+00 | 1.09E+00 | 0.038231 | 0.00449578 | 0.3251125 |
| ANGPTL4 | UD | -3.32E+00 | 2.39E+00 | 0.036153 | 0.00033399 | 3.9133743 |
| ANGPTL4 | VD | -6.49E+00 | 3.98E+00 | 0.001519 | 6.2171E-07 | 3.7091398 |
| CETP | AD | -1.24E+00 | 6.94E-01 | 0.288719 | 0.07412293 | 1.1246034 |
| CETP | AllD | -5.83E-01 | 4.76E-01 | 0.558221 | 0.21959727 | 1.4190108 |
| CETP | IHD | -6.01E-01 | 2.37E-01 | 0.548334 | 0.34480288 | 0.8720072 |
| CETP | UD | -7.76E-02 | 6.25E-01 | 0.925334 | 0.27182413 | 3.1499923 |
| CETP | VD | -1.98E-01 | 9.18E-01 | 0.82037 | 0.1357039 | 4.9593764 |
| HMGCR | AD | -5.85E-01 | 4.99E-01 | 0.557262 | 0.20974181 | 1.4805861 |
| HMGCR | AllD | -3.19E-01 | 3.45E-01 | 0.726883 | 0.36933455 | 1.4305692 |
| HMGCR | IHD | 1.01E-01 | 1.69E-01 | 1.106764 | 0.79467695 | 1.5414131 |
| HMGCR | UD | 7.49E-02 | 4.62E-01 | 1.077776 | 0.43577902 | 2.6655755 |
| HMGCR | VD | -7.63E-02 | 6.67E-01 | 0.926538 | 0.25066949 | 3.424721 |
| LPL | AD | -3.59E-01 | 8.62E-01 | 0.698612 | 0.12901998 | 3.7828133 |
| LPL | AllD | -3.14E-01 | 5.91E-01 | 0.730607 | 0.22919979 | 2.328912 |
| LPL | IHD | -1.33E+00 | 3.10E-01 | 0.264742 | 0.14415099 | 0.4862142 |
| LPL | UD | -9.89E-02 | 7.66E-01 | 0.905833 | 0.20184403 | 4.065188 |
| LPL | VD | 1.35E-01 | 1.13E+00 | 1.144537 | 0.1249552 | 10.483473 |
| NPC1L1 | AD | 8.88E-01 | 1.05E+00 | 2.43041 | 0.31215482 | 18.92296 |
| NPC1L1 | AllD | -7.36E-01 | 7.17E-01 | 0.479031 | 0.11745707 | 1.9536558 |
| NPC1L1 | IHD | -6.91E-01 | 3.58E-01 | 0.50121 | 0.24851472 | 1.0108517 |
| NPC1L1 | UD | -1.17E+00 | 9.43E-01 | 0.310367 | 0.04888523 | 1.9704856 |
| NPC1L1 | VD | 7.15E-01 | 1.43E+00 | 2.044187 | 0.12395955 | 33.710184 |
| PCSK9 | AD | 7.69E-02 | 2.89E-01 | 1.079934 | 0.61326876 | 1.9017072 |
| PCSK9 | AllD | -7.92E-02 | 2.06E-01 | 0.923855 | 0.6169545 | 1.3834218 |
| PCSK9 | IHD | -5.20E-01 | 1.08E-01 | 0.594521 | 0.48110006 | 0.7346802 |
| PCSK9 | UD | -2.20E-01 | 2.80E-01 | 0.802519 | 0.46356902 | 1.3893 |
| PCSK9 | VD | -5.54E-01 | 4.39E-01 | 0.574647 | 0.24306169 | 1.35858 |

Supplementary Table 20. One-sample MR using all available variants: Risk of dementia and ischemic heart disease in the UK Biobank.

Betas and standard errors (se) are from Cox regression models. The allele scores were calculated based on the allele frequency of the variants in the UK Biobank. Change in the risk of dementia and ischemic heart disease is per 1 mmol/L lower non-HDL cholesterol. Adjustment was for age, sex, and first 10 principal components. All variants available in CCHS+CGPS included in analyses. ANGPTL4=angiopoietin like 4; CETP=cholesteryl ester transfer protein; HMGCR= β-Hydroxy β-methylglutaryl-CoA reductase; HR=hazard ratio; LPL=lipoprotein lipase; NPC1L1=Nieman pick C1-like 1; PCSK9 = Proprotein convertase subtilisin/kexin type 9.

|  | **Vascular dementia**  Sargan statistic (p-value) | | **Unspecified dementia**  Sargan statistic (p-value) | | **Alzheimer’s disease**  Sargan statistic (p-value) | | | **All-cause dementia**  Sargan statistic (p-value) | | **Ischemic heart disease**  Sargan statistic (p-value) | |
| --- | --- | --- | --- | --- | --- | --- | --- | --- | --- | --- | --- |
| **Gene** | **CCHS+CGPS** | **UKB** | **CCHS+CGPS** | **UKB** | **CCHS+CGPS** | **UKB** | **CCHS+CGPS** | | **UKB** | **CCHS+CGPS** | **UKB** |
| **HMGCR** | 2.3  (0.31) | 1.8  (0.42) | 28  (1.0*10^-6^) | 5.245  (0.073) | 12  (0.002) | 1.846  (0.40) | 26  (1.9*10^-6^) | | 3.879  (0.14) | 9.6  (0.008) | 16.5  (0.0003) |
| **NPC1L1** | 0.59  (0.44) | 0.01  (0.91) | 26  (3*10^-7^) | 1.027  (0.31) | 1.8  (0.18) | 0.139  (0.71) | 22  (2.3*10^-6^) | | 0.003  (0.95) | 1.1  (0.29) |  |
| **PCSK9** | 1.9  (0.75) | 1.4  (0.84) | 2.5  (0.65) | 4.45  (0.35) | 0.32  (0.99) | 9.74  (0.05) | 0.44  (0.98) | | 2.52  (0.64) | 7.7  (0.10) | 9.05  (0.06) |
| **ANGPTL4** | NA | NA | NA | NA | NA | NA | NA | | NA | NA | NA |
| **LPL** | 2.0  (0.6) | 3.1  (0.37) | 13  (0.004) | 0.682  (0.88) | 0.66 (0.88) | 6.074  (0.11) | 4.5  (0.21) | | 3.432  (0.33) | 3.5  (0.32) | 3.5  (0.32) |
| **CETP** | 6.5  (0.48) | 3.3  (0.85) | 80  (1.16*10^-14^) | 7.8  (0.35) | 15  (0.04) | 3.334  (0.85) | 55  (1.5*10^-9^) | | 4.966  (0.66) | 13  (0.06) | 6.2  (0.52) |

Supplementary Table 21. Sargan Statistics for genetic instruments with more than one variant used in sensitivity analyses including all variants.

Sargan Statistics were calculated for drug targets with more than one genetic instrument using the ivreg package (<https://cran.r-project.org/web/packages/ivreg/vignettes/ivreg.html>). ANGPTL4=angiopoietin like 4; CETP=cholesteryl ester transfer protein; CCHS = Copenhagen City Heart Study; CGPS=Copenhagen General Population Study; HMGCR= β-hydroxy β-methylglutaryl-CoA reductase; HR=hazard ratio; LPL=lipoprotein lipase; NPC1L1=Nieman pick C1-like 1; PCSK9 = Proprotein convertase subtilisin/kexin type 9..

**
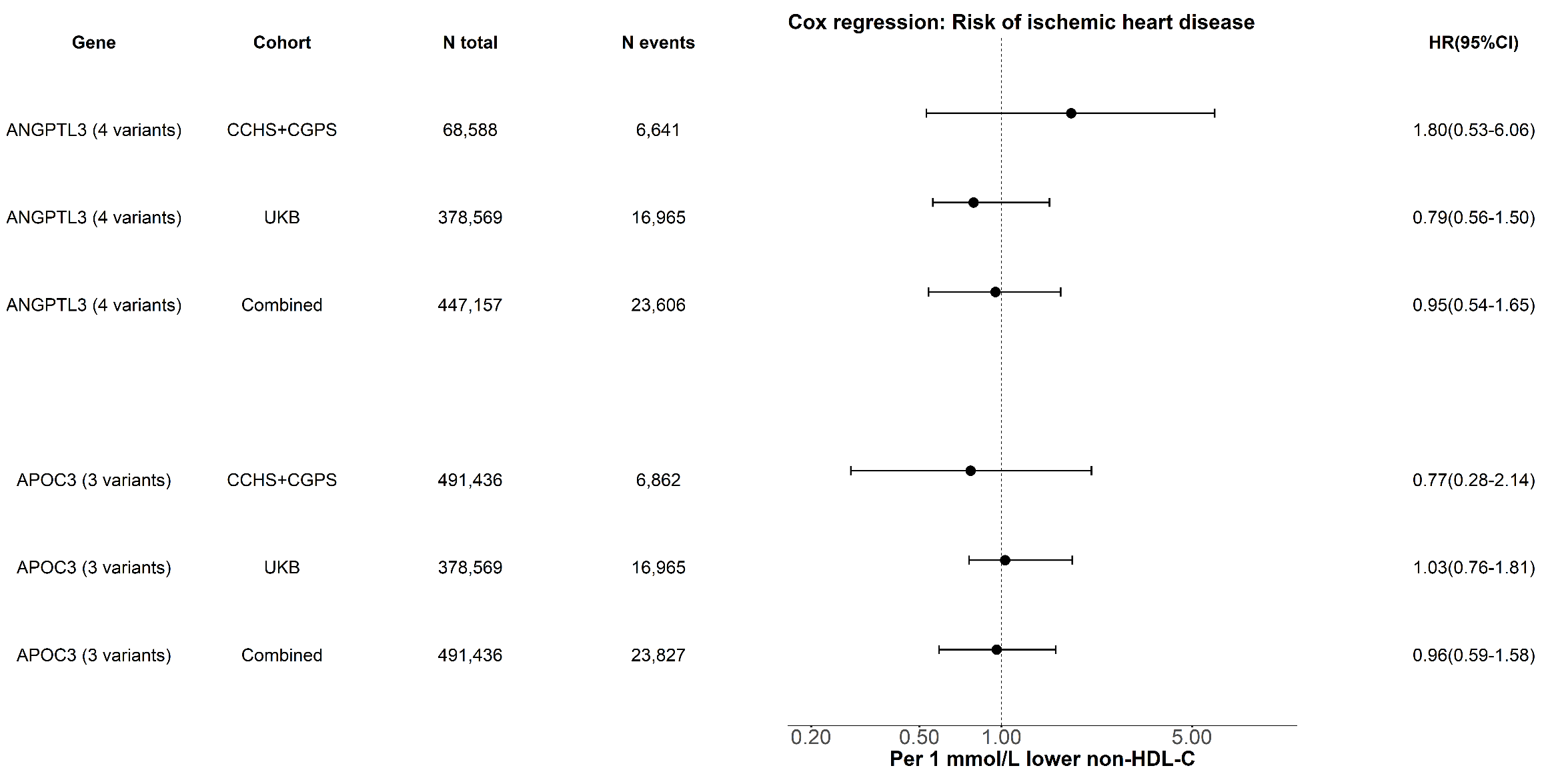
**Supplementary Figure 1. Cox regression: Risk of ischemic heart disease in CCHS+CGPS and UKB. Variants included rs138326449, rs147210663, and rs76353203 (APOC3), and rs1430463625, rs1468742046, rs267606655, and rs372257803 (ANGPTL3). Betas and standard errors (se) are from Cox regression models. The allele scores were calculated based on the allele frequency of the variants in the CCHS+CGPS and the UK Biobank. Change in the risk of ischemic heart disease is per 1 mmol/L lower non-HDL cholesterol. Q= 0.24 for APOC3, and 1.36 for ANGPTL3. F statistics were 29 and 231 for APOC3, and 21 and 207 for ANGPTL3 in UK Biobank Adjustment was for age and sex in CCHS+CGPS and for age, sex, and first 10 principal components in UK Biobank. APOC3= apolipoprotein C3; ANGPTL3=angiopoietin like 3; CI=confidence interval; HMGCR= β-Hydroxy β-methylglutaryl-CoA reductase; HR=hazard ratio.

**
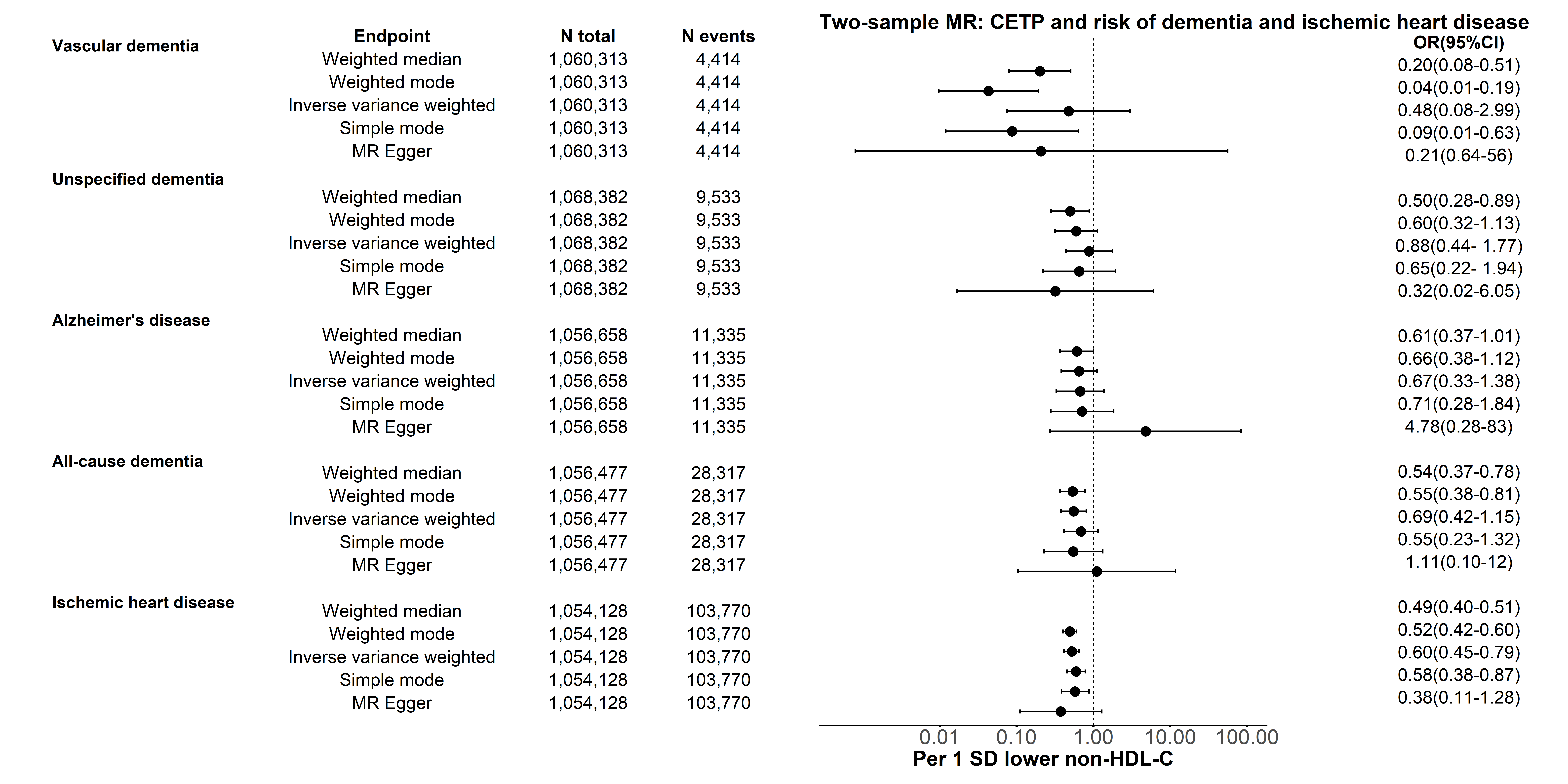
**Supplementary Figure 2. Genetic variation in *CETP* and risk of dementia and ischemic heart disease. Based on meta-analyses of results from Copenhagen City Heart Study, Copenhagen General population Study, UK Biobank, FinnGen and Global Lipids Genetics Consortium. Analyses were performed using the “TwoSampleMR” R package. Heterogeneity tests for meta-analyses: Q=0.49 to 21.15 for vascular dementia, 0.48 to 16.07 for unspecified dementia, 0.50 to 9.19 for Alzheimer’s disease, 0.30 to 17.31 for all-cause dementia, and 0.37 to 19.98 for ischemic heart disease. Mean F statistic for *CETP* was 219. CETP=cholesteryl ester transfer protein; Supplementary Figure 3. Genetic variation in *PCSK9* and risk of dementia and ischemic heart disease. Based on meta-analyses of results from Copenhagen City Heart Study, Copenhagen General population Study, UK Biobank, FinnGen and Global Lipids Genetics Consortium. Analyses were performed using the “TwoSampleMR” R package. Heterogeneity tests for meta-analyses: Q=0.54 to 1.45 for vascular dementia, 0.31 to 2.22 for unspecified dementia, 0.28 to 2.68 for Alzheimer’s disease, 0.18 to 0.67 for all-cause dementia, and 0.09 to 2.11 for ischemic heart disease. Mean F statistic for *PCSK9* was 661. PCSK9 = Proprotein convertase subtilisin/kexin type 9.**
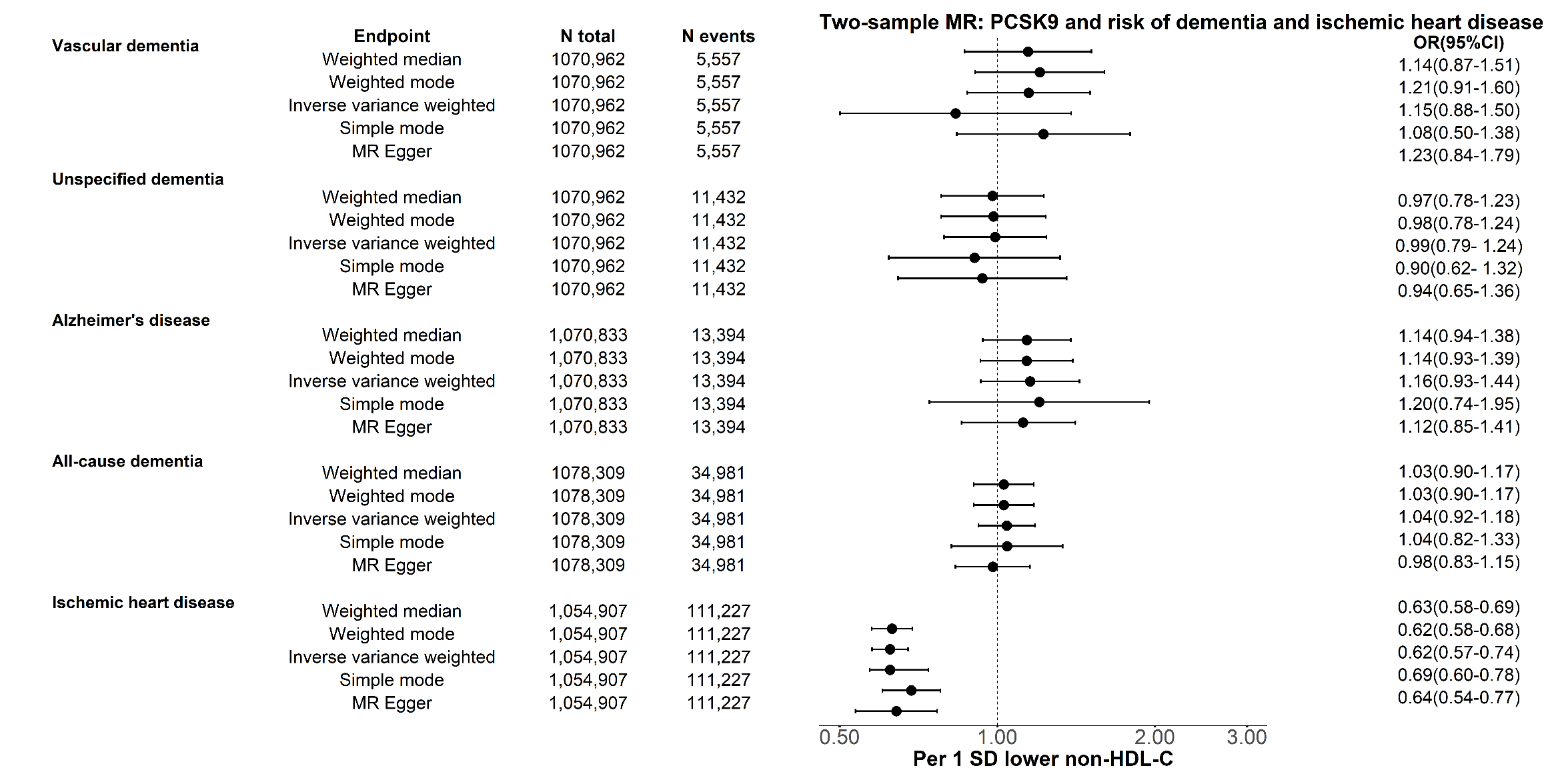
**

**
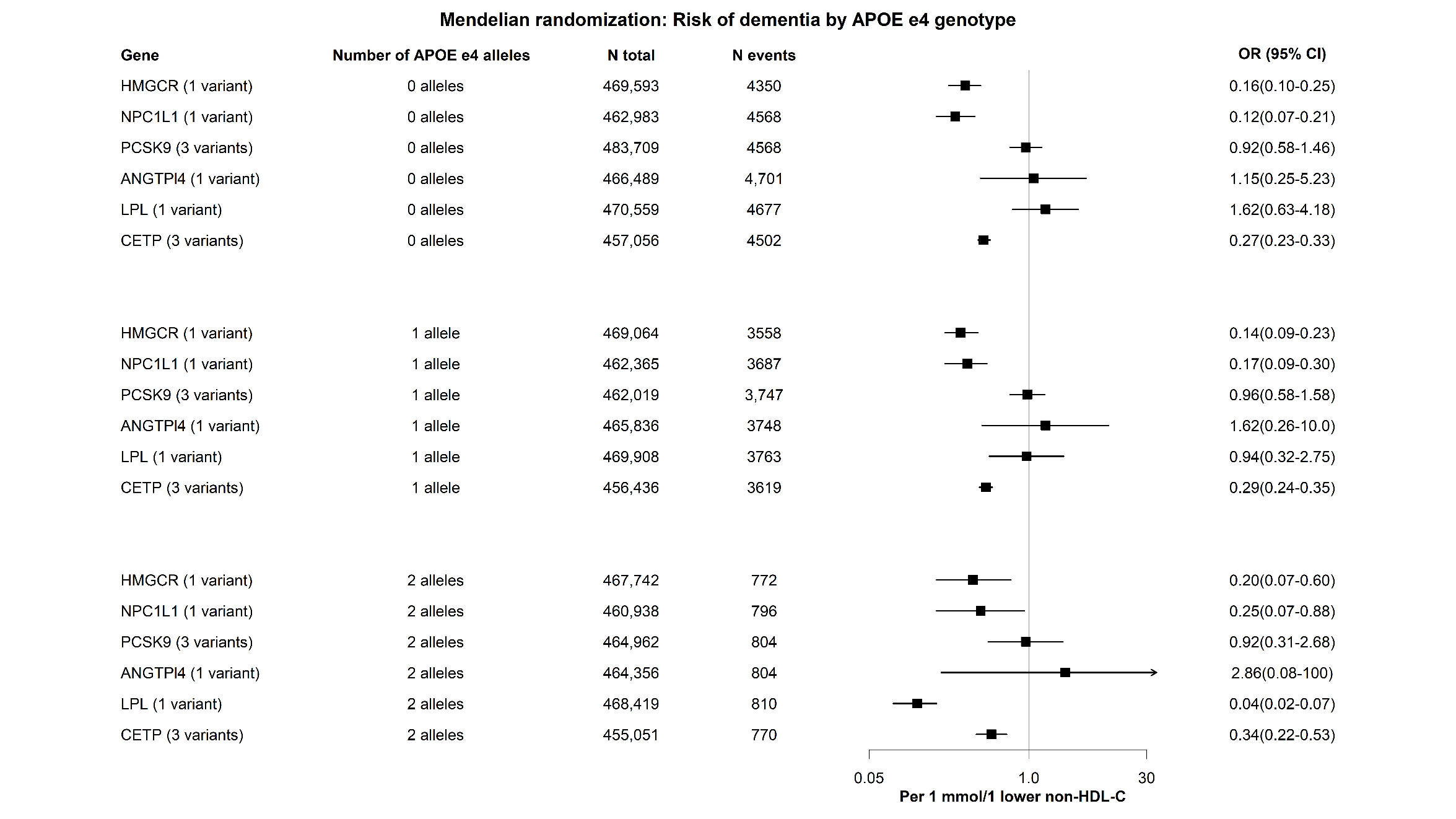
**Supplementary Figure 4. Mendelian randomization: Risk of dementia by APOE e4 genotype in CHHS+CGPS and UK Biobank. Odds ratios (ORs) and 95% confidence intervals are from one-sample MR analyses. The allele scores were calculated based on the allele frequency of the variants in the CCHS+CGPS and UK Biobank. Change in the risk of vascular-related dementia is per 1 mmol/L lower LDL cholesterol for *HMGCR* and *NPC1L1*, per halving in triglycerides for *ANGPTL4* and *LPL*, and per 1 mmol/L lower non-HDL cholesterol for *CETP*. The Mendelian randomization estimates were derived using the “OneSampleMR” package. Meta-analyses were performed using the “meta” R package. Heterogeneity tests for meta-analyses: Q=0.21 to 2.48 for HMGCR, 0.01 to 1.51 for NPC1L1, 0.00 to 2.39 for PCSK9, 0.83 to 2.85 for ANGPTL4, 0.22 to 4.78 for LPL, and 0.00 to 3.50 for CETP. Dementia subtype was all-cause dementia (vascular dementia, unspecified dementia, and Alzheimer’s disease) subdivided by *APOE* ɛ4 genotype (wildtype, heterozygotes, or homozygotes). CI= confidence interval; OR=odds ratio; CCHS=Copenhagen City Heart Study; CGPS=Copenhagen General Population Study. ANGPTL4=angiopoietin like 4; CETP=cholesteryl ester transfer protein; CPH = Copenhagen studies; CI=confidence interval; HMGCR= β-Hydroxy β-methylglutaryl-CoA reductase; HR=hazard ratio; LPL=lipoprotein lipase; NPC1L1=Nieman pick C1-like 1; PCSK9 = Proprotein convertase subtilisin/kexin type 9.

**
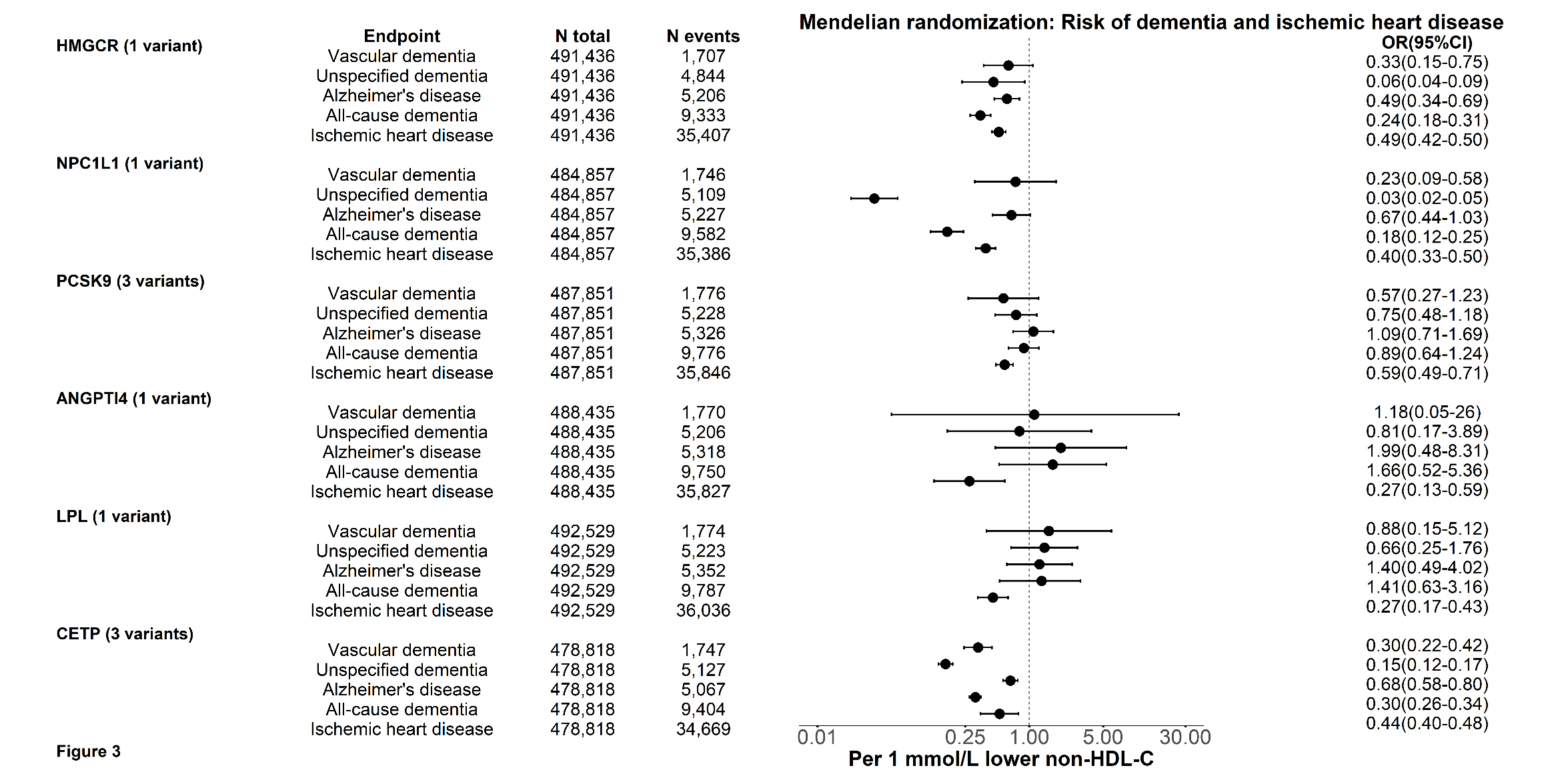
**Supplementary Figure 5. Mendelian randomization using all variants: Risk of dementia and ischemic heart disease in CCHS+CGPS and UK Biobank. Betas and standard errors (se) are from one-sample MR using the ‘OneSampleMR’ package. Heterogeneity tests for meta-analyses: Q=0.38 to 7.88 for HMGCR, 0.56 to 5.92 for NPC1L1, 0.00 to 0.18 for PCSK9, 1.90 to 3.28 for ANGPTL4, 0.11 to 0.41 for LPL, and 1.02 to 7.91 for CETP. The genetic variants were used as individual instruments. Change in the risk of dementia and ischemic heart disease is per 1 mmol/L lower non-HDL cholesterol. Adjustment was for age and sex in CCHS+CGPS and for age, sex, and first ten principal components in UK biobank. All variants available in CCHS+CGPS included in analyses. ANGPTL4=angiopoietin like 4; CETP=cholesteryl ester transfer protein; CPH = Copenhagen studies; CI=confidence interval; HMGCR= β-Hydroxy β-methylglutaryl-CoA reductase; HR=hazard ratio; LPL=lipoprotein lipase; NPC1L1=Nieman pick C1-like 1; PCSK9 = Proprotein convertase subtilisin/kexin type 9.


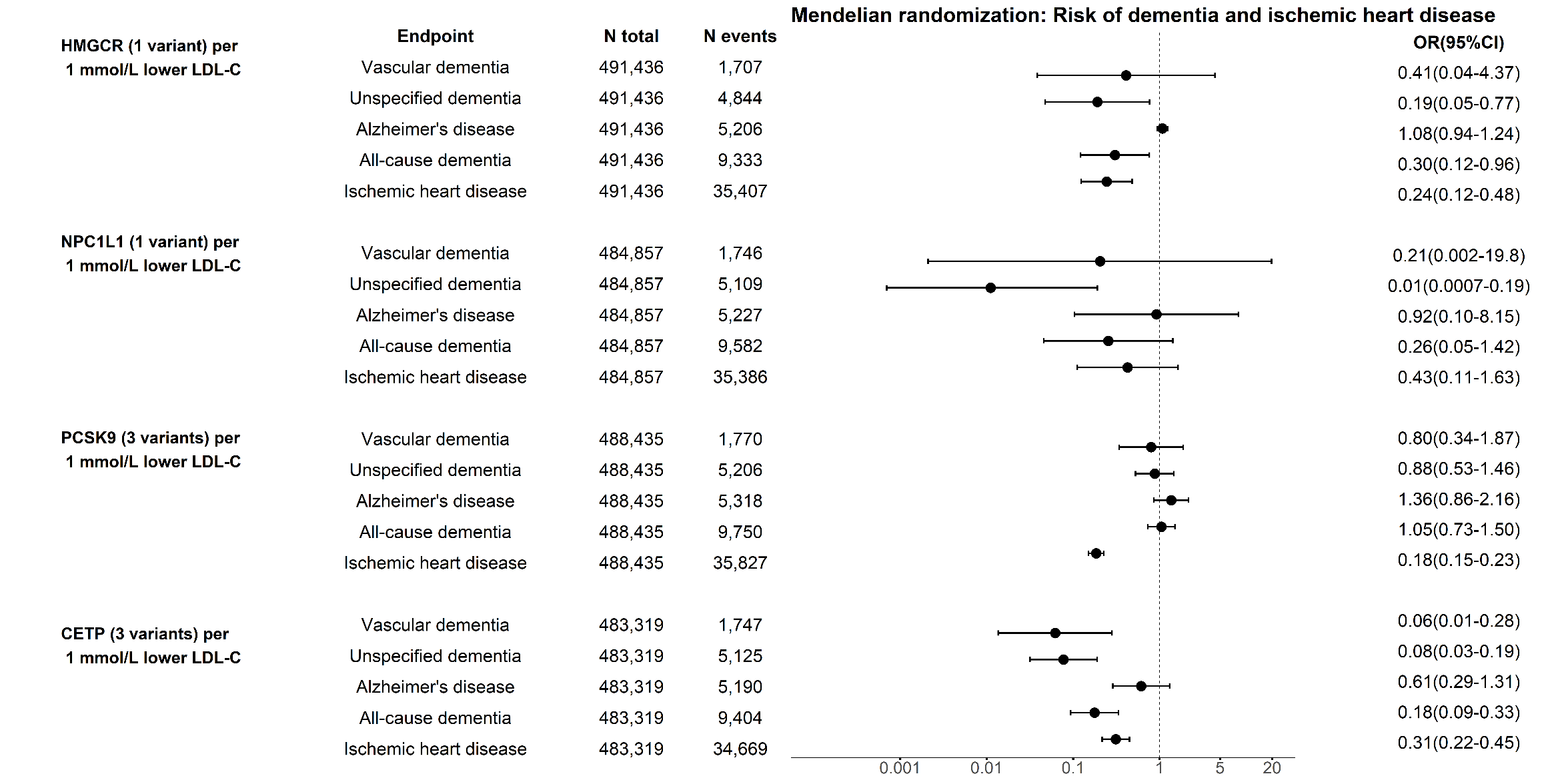


Supplementary Figure 6. Mendelian randomization: Risk of dementia and ischemic heart disease in CCHS+CGPS and UK Biobank. Odds ratios and 95% confidence intervals are from one-sample MR using the ‘OneSampleMR’ package. Heterogeneity tests for meta-analyses: Q=0.21 to 2.48 for HMGCR, 0.01 to 1.51 for NPC1L1, 0.00 to 0.18 for PCSK9, and 0.00 to 3.50 for CETP. The genetic variants were used as individual instruments. Change in the risk of dementia and ischemic heart disease is per 1 mmol/L lower LDL cholesterol, per halving in triglycerides, and per 1 mmol/L lower non-HDL cholesterol. Adjustment was for age and sex in CCHS+CGPS and for age, sex, and first ten principal components in UK biobank. All variants available in CCHS+CGPS included in analyses. CETP=cholesteryl ester transfer protein; CPH = Copenhagen studies; CI=confidence interval; HMGCR= β-Hydroxy β-methylglutaryl-CoA reductase; HR=hazard ratio; NPC1L1=Nieman pick C1-like 1; PCSK9 = Proprotein convertase subtilisin/kexin type 9.

**
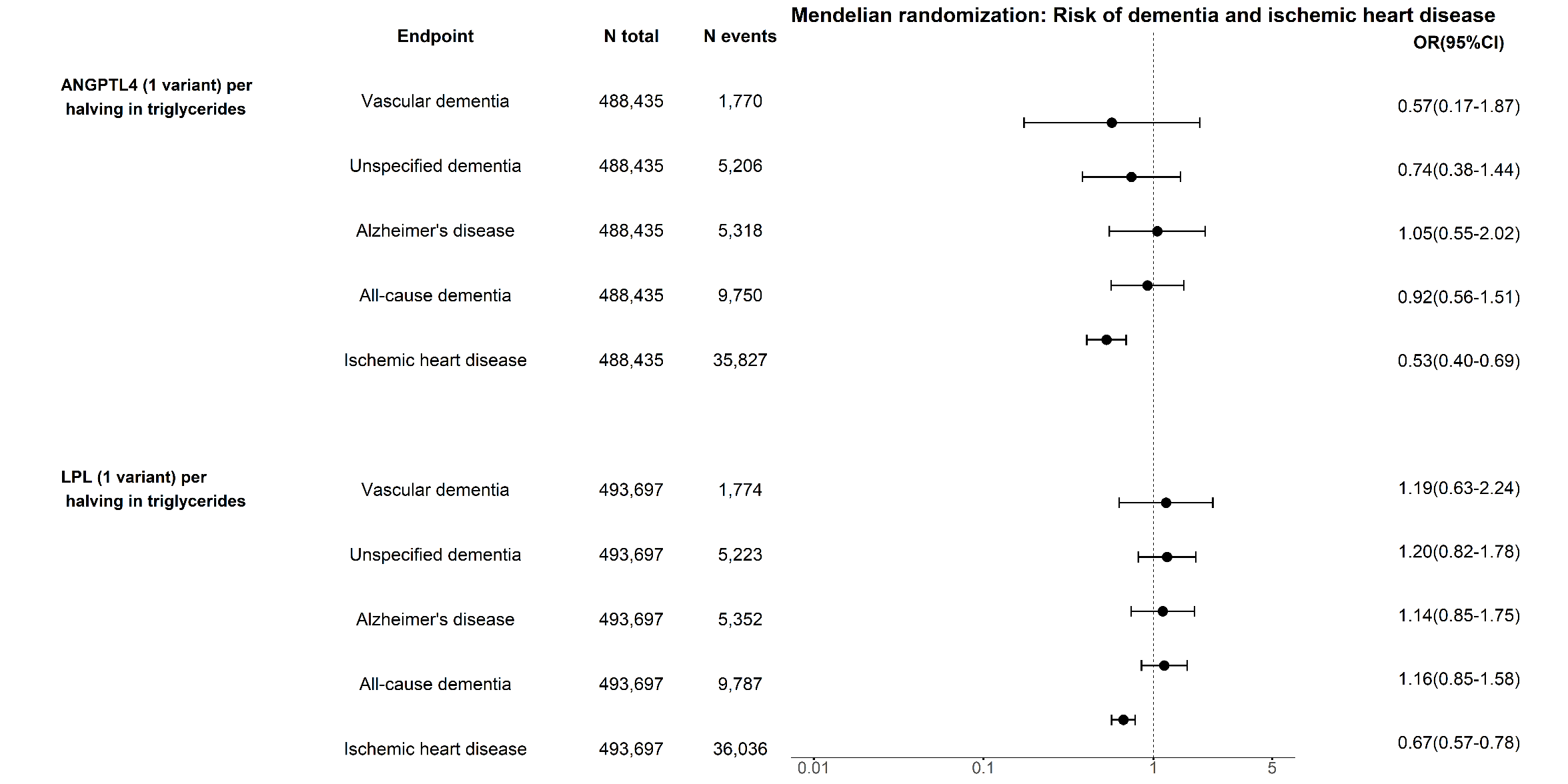
**Supplementary Figure 7. Mendelian randomization: Risk of dementia and ischemic heart disease in CCHS+CGPS and UK Biobank. Odds ratios and 95% confidence intervals are from one-sample MR using the ‘OneSampleMR’ package. Heterogeneity tests for meta-analyses: Q=0.83 to 2.85 for ANGPTL4, 0.22 to 4.78 for LPL. The genetic variants were used as individual instruments. Change in the risk of dementia and ischemic heart disease is per halving in triglycerides. Adjustment was for age and sex in CCHS+CGPS and for age, sex, and first ten principal components in UK biobank. All variants available in CCHS+CGPS included in analyses. ANGPTL4=angiopoietin like 4; CPH = Copenhagen studies; CI=confidence interval; HR=hazard ratio; LPL=lipoprotein lipase.

**
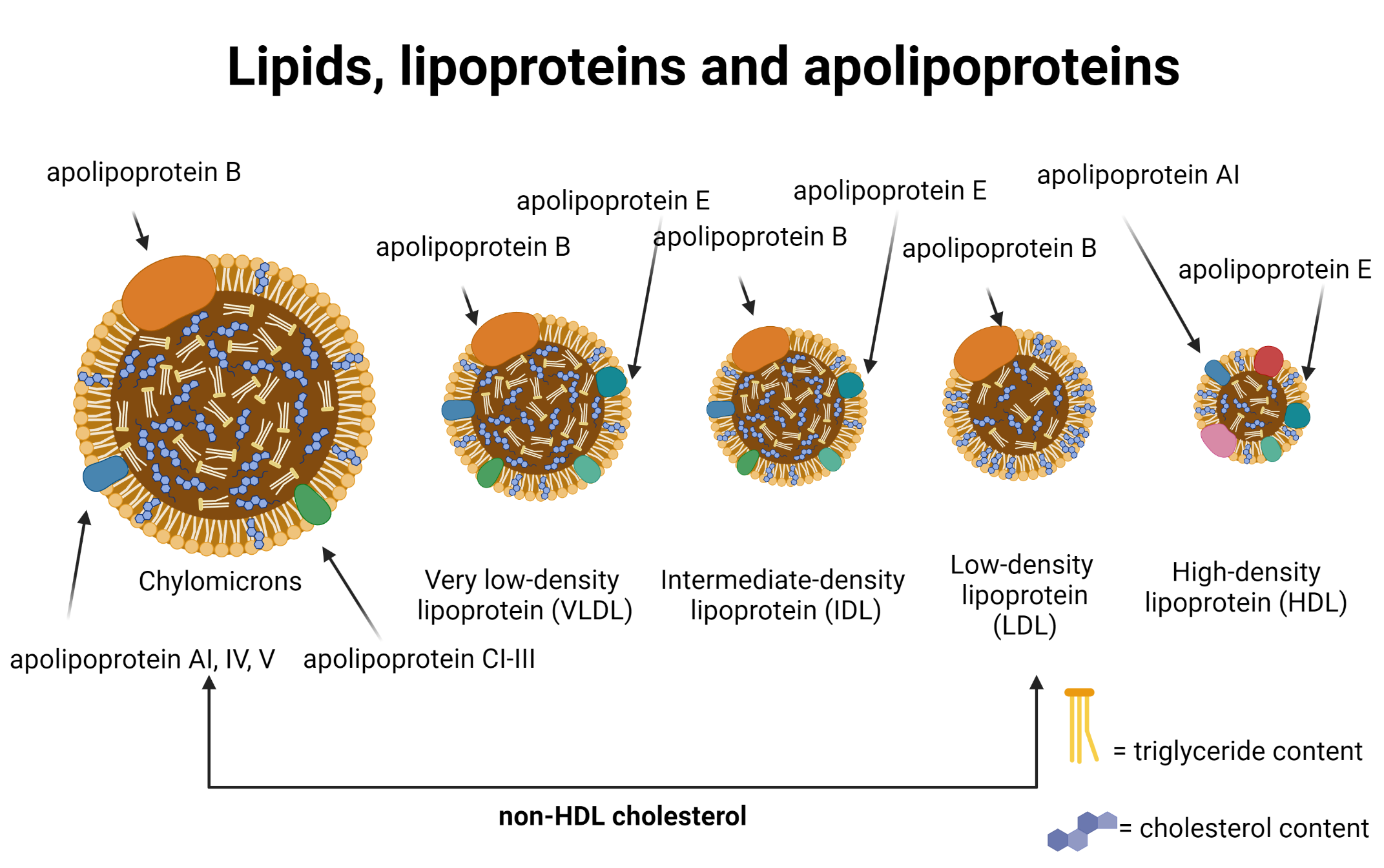
**

Supplementary Figure 8. Illustration of plasma lipids, lipoproteins, and apolipoproteins. Only some apolipoproteins are shown. Adapted from [1]. Created with Biorender.com.

# References

1. Nordestgaard LT, Christoffersen M, Frikke-Schmidt R. Shared Risk Factors between Dementia and Atherosclerotic Cardiovascular Disease. Int J Mol Sci. 2022;23:9777.
